# IRIS-QResNet: A Quantum-Inspired Deep Model for Efficient Iris Biometric Identification and Authentication

**DOI:** 10.3390/s26010121

**Published:** 2025-12-24

**Authors:** Neama Abdulaziz Dahan, Emad Sami Jaha

**Affiliations:** Department of Computer Science, Faculty of Computing and Information Technology, King Abdulaziz University, Jeddah 21589, Saudi Arabia; ejaha@kau.edu.sa

**Keywords:** iris recognition, biometrics, identification, authentication, quantum-inspired, custom ResNet, quanvolutional, computer vision, hybrid quantum classical model

## Abstract

**Highlights:**

- IRIS-QResNet, a custom ResNet model enhanced with a quanvolutional layer for more accurate iris recognition that uses few samples per subject without applying augmentation.
- IRIS-QResNet proves its ability for efficient biometric authentication by consistently achieving superior accuracy and generalization across four benchmark datasets.

**What are the main findings?**

- Compared with IResNet, the traditional baseline, IRIS-QResNet model significantly improves recognition accuracy and robustness, even in small-sample, augmentation-free settings.
- Across multiple iris datasets, IRIS-QResNet strengthens multilayer feature extraction, resulting in measurable performance gains of up to 16.66% in identification accuracy.

**What are the implications of the main findings?**

- By effectively integrating quantum-inspired layers into classical deep networks, higher discriminative power and data efficiency can be achieved, reducing dependence on large training datasets and data augmentation.
- These results open the path toward scalable and sustainable AI solutions for biometric systems, establishing a viable bridge between conventional and emerging quantum machine learning architectures.

**Abstract:**

Iris recognition continues to pose challenges for deep learning models, despite its status as one of the most reliable biometric authentication techniques. These challenges become more pronounced when training data is limited, as subtle, high-dimensional patterns are easily missed. To address this issue and strengthen both feature extraction and recognition accuracy, this study introduces IRIS-QResNet, a customized ResNet-18 architecture augmented with a quanvolutional layer. The quanvolutional layer simulates quantum effects such as entanglement and superposition and incorporates sinusoidal feature encoding, enabling more discriminative multilayer representations. To evaluate the model, we conducted 14 experiments on the CASIA-Thousands, IITD, MMU, and UBIris datasets, comparing the performance of the proposed IRIS-QResNet with that of the IResNet baseline. While IResNet occasionally yielded subpar accuracy, ranging from 50.00% to 98.66%, and higher loss values ranging from 0.1060 to 2.0640, comparative analyses showed that IRIS-QResNet consistently outperformed it. IRIS-QResNet achieved lower loss (ranging from 0.0570 to 1.8130), higher accuracy (ranging from 66.67% to 99.55%), and demon-started improvement margins spanning from 0.1870% in the CASIA End-to-End subject recognition with eye-side to 16.67% in the MMU End-to-End subject recognition with eye-side. Loss reductions ranged from 0.0360 in the CASIA End-to-End subject recognition without eye-side to 1.0280 in the UBIris Non-End-to-End subject recognition. Overall, the model exhibited robust generalization across recognition tasks despite the absence of data augmentation. These findings indicate that quantum-inspired modifications provide a practical and scalable approach for enhancing the discriminative capacity of residual networks, offering a promising bridge between classical deep learning and emerging quantum machine learning paradigms.

## 1. Introduction

**Figure 1:**
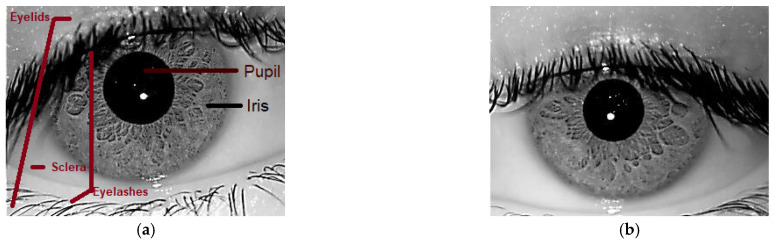
The right and left eyes of subject no. 53 of the IITD dataset, showing the distinctiveness of the irises is even among the two eyes of the same person. (**a**) The right eye; (**b**) the left eye.

Safe and trustworthy identity authentication has grown to be a major worldwide concern. In a variety of fields, today, including banking digital platforms, healthcare, and border control, people are required to present identification. Traditional password-based systems are becoming increasingly ineffective because they are vulnerable to theft, duplication, and human error. A safer substitute, however, is provided by biometric technologies, which are based on distinct physiological or behavioral characteristics [1]. The International Organization for Standardization (ISO) and the International Electrotechnical Commission (IEC) [2] define biometric systems as automated procedures that uniquely identify people based on behavioral or physiological traits. In contrast to behavioral modalities, which include voice, gait, and typing dynamics, physiological modalities include iris patterns, facial geometry, and fingerprints [3]. Iris recognition is highly unique, stable, informative, safe, and contactless [4]. The iris of humans, as shown in Figure 1, has special textural structures [5] that are incredibly intricate, persistent throughout life, and even different between the two eyes of an individual [6]. The iris largely remains unchanged after early childhood and even after death [7], in contrast to fingerprints that may deteriorate due to physical labor or facial features that can change with aging, cosmetic makeup, or surgeries. Additionally, non-contact hygienic and forgery-resistant iris-based systems have remarkably low rates of false acceptance and rejection [8]. Therefore, the iris is regarded as one of the most reliable physiological characteristics for extensive identity authentication [9,10].

The three primary phases of a handcrafted iris recognition pipeline are segmentation, feature extraction, and matching [11]. The iris region is often segmented to eliminate occlusions from the eyelids and lashes after obtaining a high-quality eye image. After the iris texture has been segmented, discriminative features are extracted and compared for identification or verification [11]. Early methods used handcrafted features like Local Binary Patterns (LBP) [12], Gabor filters [13], and wavelet transforms [14], which were frequently coupled with conventional classifiers like Support Vector Machines (SVMs) or K-Nearest Neighbors (k-NN) [15]. These techniques performed well in controlled settings, but struggled with noise and nonlinear texture variations, which limited their robustness and cross-sensor generalization.

Iris recognition has advanced significantly since the emergence of deep learning. Manual feature engineering is rapidly becoming no longer necessary because Deep Neural Networks (DNNs) and Residual Networks (ResNets) can automatically learn hierarchical and highly discriminative features straight from raw iris images [4,11]. ResNets can achieve better accuracy and robustness by capturing both local and global iris textural patterns using residual skip connections and very deep convolutional blocks [16]. When compared to the basic Conventional Neural Networks (CNNs), their end-to-end architecture simplifies the procedure and produces better benchmark results [4,17]. Deep models, however, have several drawbacks: (1) they need large training datasets in order to generalize well [16,18], (2) using small iris datasets, which typically contain only five to ten samples per class, may result in overfitting and decreased accuracy [19], (3) training and inference are computationally costly, which limits their use on devices with limited resources [20], and (4) end-to-end systems may unintentionally capture/rely on irrelevant information like scleral or eyelid features, while robust iris localization is still a challenge [21,22]. These limitations are particularly problematic for iris recognition because acquiring large-scale iris datasets is constrained by privacy regulations, collection costs, and the requirement for specialized near-infrared imaging equipment. Moreover, conventional data augmentation techniques, such as rotation, scaling, and brightness adjustment, merely increase sample quantity without fundamentally enriching the feature representation space. This approach fails to address the core challenge: how to extract maximally discriminative features from the limited available data while avoiding the capture of spurious, non-iris patterns.

The intricate, multiscale texture patterns of the iris present unique challenges for conventional CNNs. Although CNN-based approaches demonstrate strong performance in iris recognition when trained on large datasets, they face clear limitations in small-sample regimes due to: (1) localized receptive fields that may fail to capture long-range spatial dependencies between distant iris features [23] (2) fixed kernel sizes that constrain multiscale feature extraction [24], and (3) high sensitivity to limited training data [25]. These constraints are particularly critical in iris recognition, where large-scale data collection is restricted by privacy concerns and specialized acquisition requirements. Quantum-inspired transformations offer a mechanism for addressing these challenges [26]. Specifically, quanvolutional layers employ parameterized operations derived from principles of quantum superposition and entanglement to project classical image data into exponentially larger Hilbert spaces [27]. Superposition enables the simultaneous evaluation of multiple feature combinations, allowing the model to examine numerous texture patterns in parallel, a capability that becomes especially valuable in data-scarce scenarios, where exhaustive feature exploration is infeasible [28]. Additionally, entanglement-based transformations can encode long-range spatial dependencies and non-local correlations within iris textures that standard convolutions, constrained by limited receptive fields, cannot sufficiently represent [29]. These operations enrich the representational space by introducing additional degrees of freedom and cross-channel correlations without requiring quantum hardware [30,31]. Unlike data augmentation, which solely increases sample quantity, quanvolutional layers fundamentally expand the model’s representational capacity [30]. By projecting features into higher-dimensional spaces through parameterized quantum transformations, these layers facilitate the discovery of discriminative iris patterns that are beyond the expressive reach of classical convolutional filters [31]. This enhancement allows the network to focus more effectively on genuine iris texture information, reducing reliance on irrelevant scleral or eyelid features. Recent studies have reported that quanvolutional layers improve generalization and pattern separability [32,33], with increased robustness to noise and cross-dataset variability [28,29,34]. Despite these promising developments, the use of quantum-inspired techniques in iris recognition remains largely unexplored. Given the natural correspondence between the high-dimensional structure of iris textures and the expanded representational potential of quantum-inspired transformations, this research direction warrants systematic investigation.

By incorporating quanvolutional layers into residual blocks, the hybrid ResNet-18 framework proposed in this study (IRIS-QResNet) seeks to close that gap. Moreover, instead of relying on synthetic sample expansion, the proposed IRIS-QResNet directly improves feature richness by enriching the representational space of the input, rather than merely increasing its quantity. This strategy leverages quantum-inspired feature mixing to extract fine-grained iris textures without introducing augmentation-induced distortions, offering particular advantages in extremely small-sample regimes. From small and varied datasets, this integration enables efficient deep feature extraction with enhanced generalization, while preserving computational efficiency. The suggested approach aims for high accuracy and robustness even with small sample sizes per class, in contrast to standard deep models that primarily rely on data augmentation or require massive datasets. The contributions of this paper are threefold:

1. A hybrid iris recognition framework that supports both End-to-End and non-End-to-End modes and it is generalizable and applicable to a variety of datasets, varying in sizes, i.e., tiny, small, and mid-size.
2. A unique customized ResNet-18 architecture that can manage datasets with different dimensions and forms, including very small-sample regimes (e.g., five to ten images per class) without the need for data augmentation.
3. An experimental examination of quantum-inspired improvements by the proposed IRIS-QResNet model that is methodical and involves integrating quanvolutional layers and assessing it on four benchmark datasets, along with comparative analysis to measure gains in accuracy, robustness, and loss reduction.

The remainder of the paper is organized as follows: Section 2 describes the background and related works. Section 3 introduces the proposed custom ResNet-18 model with the quanvolutional layer. Section 4 is for setting up the experiment. Section 5 discusses the experiments conducted together with their results and comparative evaluations. Finally, conclusions are drawn in Section 6.

## 2. Background and Related Work

This section reviews the generations of iris recognition systems from basic handcrafted techniques to modern deep CNN and quantum-inspired models.

### 2.1. Handcrafted Iris Recognition Approaches

**Figure 2:**
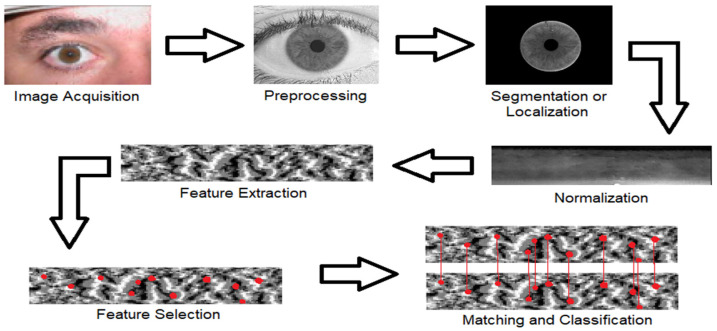
Overview of the handcrafted iris recognition pipeline. Key processing stages follow the approaches outlined in [36,37].

The history of iris recognition closely resembles the evolution of biometric systems generally. Due to the iris’s uniqueness, stability, and rich texture patterns, researchers have turned their attention to it as a significant modality for secure identity authentication in digital and governmental applications [35]. The early efforts of iris recognition pipelines [36,37,38], as shown in Figure 2, include iris image acquisition, preprocessing, segmentation, normalization, feature extraction, feature selection (represented as red dots in the figure), matching of the selected features, and classification of the recognized subjects. Since the accuracy of the system was directly influenced by the discriminative power of the extracted features, the feature extraction stage was the most important. Earlier systems employed handcrafted features based on mathematical transformations such as wavelet transforms [14], which provided multi-resolution analysis, and Gabor filters [13], which modeled texture frequency and orientation. Additionally, Local Binary Patterns (LBP) [13] offered straightforward yet useful micro-texture descriptors. Shallow classifiers, such as SVM or k-NN, were commonly used in conjunction with these features [15]. Because of their interpretability and computational efficiency, these methods performed well under controlled imaging conditions. Nevertheless, several restrictions surfaced [4,11]. Handcrafted features had a fixed descriptive power once they were designed, making them less flexible to change datasets or environmental conditions [39]. Their performance might drastically decline in unrestricted settings with noise, occlusions, or lighting shifting. Furthermore, these approaches repeatedly worked in low-dimensional feature spaces, preventing them from capturing hierarchical and highly nonlinear information in iris textures. Precise segmentation was also crucial for accuracy, where mistakes in boundary localization greatly reduced performance [4,11]. It was evident by the late 2000s that handcrafted pipelines lacked generalization capabilities despite being effective and interpretable. Deep learning approaches were made possible by the growing efforts to devise techniques that could automatically learn robust nonlinear representations.

### 2.2. Deep CNN-Based Iris Recognition Approaches

Iris recognition was revolutionized by deep learning [40], which removed the need for manual techniques by enabling models to learn discriminative representations straight from data [41,42]. According to Yin et al. [4], because of their capacity to take advantage of spatial hierarchies in images, CNNs became the most popular deep learning architecture. Manual feature engineering is eliminated by end-to-end CNN-based systems, which feed raw iris images into the network, and an embedding or classification label is the output. Comparison with handcrafted techniques, CNNs significantly decreased false acceptance and rejection rates [4,8]. Multimodal frameworks enhanced robustness in difficult situations by combining iris data with other biometric modalities like face or fingerprint [10], transferring learning of pre-trained networks from sizable natural image datasets [18], and enforcing hybrid CNN-transformer models that capture long-range dependencies [40]. ResNets are a well-known subclass of CNN architecture made to address issues with feature degradation and vanishing gradients in very deep networks. They are especially beneficial in improving feature learning and robustness by maintaining the hierarchical convolutional structure, while adding skip connections. However, challenges persist despite these pros, as several iris datasets include only a few samples per subject, while CNNs require large datasets to generalize effectively. When models memorize training data instead of acquiring transferable patterns, this may often result in overfitting. Augmenting data (e.g., rotation, scaling, and illumination changes) [16,18] can introduce artifacts and provide only a limited amount of improvement. Computationally demanding deep networks require a lot of computing power [4,11], where training and deployment require powerful GPUs [43]. End-to-end CNNs may also unintentionally pick up unrelated features (e.g., damaging cross-dataset generalization or eyelid textures of sclera veins) [4]. On the other hand, iris localization is still challenging across a variety of sensors and settings [39,44]. Lack of data, high computational cost, poor localization, and degraded cross-sensor robustness are among the problems that highlight the need for alternative paradigms that preserve CNNs’ learning capabilities while enhancing efficacy and versatility. Quantum-inspired computing is a promising avenue to pursue.

### 2.3. Quantum-Inspired Image Classification Approaches and Remaining Gaps

Recent applications of quantum computing ideas to solve classical (non-quantum) machine learning have led to quantum-inspired methodologies. While true quantum computers are still in the Noisy Intermediate-Scale Quantum (NISQ) stage [45], models inspired by quantum technology use standard hardware to simulate quantum behavior [26,46]. Such models improve pattern separability without the use of quantum processors by leveraging high-dimensional feature mappings that are comparable to those of quantum systems. A quanvolutional (quantum convolutional) layer is inspired by quantum computing concepts added to a DNN model for enhancement reasons [33,47]. It splits an image into patches, encodes each patch as a quantum state, performs unitary transformations, and then projects the output back into classical space. By enriching feature representations, this process enables easier linear separation of intricate or overlapping patterns. Research on general image classification demonstrates that quanvolutional layers can improve robustness to noise and cross-domain variations, while improving generalization with fewer parameters [26,33,47]. Integration into current pipelines is allowed by compatibility with CNN architectures [33,48,49]. However, there is still much to learn about the applications of quantum-inspired techniques to biometric recognition, particularly iris recognition. Simple tasks like classifying flowers or numbers have been among the focus of previous research [48,50]. Research on the interaction of quanvolutional layers with biometric [51,52,53], iris-specific problems, like small datasets, cross-sensor variability, or imprecise localization, is scarce if it exists at all; to the best of our knowledge, few articles are available on biometrics, face-specific, but none about iris. Furthermore, little is known about how quantum-inspired encoding and data augmentation interact. Basically, quantum-encoded data, which is transformed using a quantum transformer such as the Quantum Fourier Transformer (QFT), may be distorted by the augmentation transformer, thereby reducing performance rather than increasing it [54]. The absence of cross-dataset evaluation in earlier research work is another significant drawback. It is challenging to evaluate generalization in real-world scenarios with numerous sensors and diverse populations, as many experiments rely on a single dataset.

To fill these research gaps, this study presents IRIS-QResNet as a customized ResNet-18 framework that incorporates quanvolutional layers into its residual blocks. The suggested hybrid architecture preserves compatibility with ResNet architecture while increasing feature extraction efficiency and generalization on augmentation-free small-sample data. Four different iris datasets are evaluated in fourteen experiments that directly contrast baseline and hybrid configurations. This study bridges the gap between CNN-based and quantum-inspired paradigms in biometric systems by being one of the first thorough examinations of quantum-inspired deep learning for iris recognition.

## 3. IRIS-QResNet: The Proposed Model

To address the aforementioned issues, we propose IRIS-QResNet, a system customized for iris recognition purposes that is enhanced with a quantum-inspired layer (also known as quanvolutional layer), as its name suggests. The core design of our proposed model (IRIS-QResNet) is the baseline model (IResNet), as we denote throughout the rest of this paper. Though it shares structural similarities with the standard ResNet-18, we customized it for iris biometrics to function well even on high-variation low-sample datasets, such as those with as few as five images per class. In contrast to standard ResNet-18 configurations tailored for extensive natural image datasets (e.g., ImageNet), IResNet integrates training and architectural adaptation to improve data efficiency and better suit the subtle intra-class variability and fine-grained textures found in iris patterns.

### 3.1. IResNet: The Baseline Model

**Figure 3:**
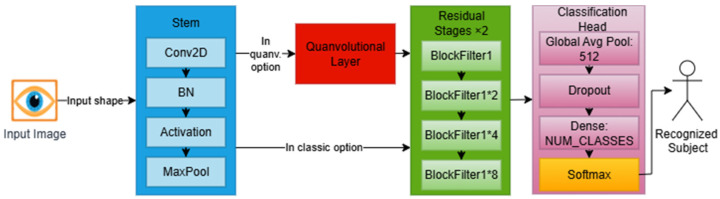
IRIS-QResNet conceptual model, showing where the quanvolutional layer is installed to extend our baseline IResNet model to the proposed quantum-inspired counterpart.

The conventional ResNet-18 architecture serves as the core of our baseline model (IResNet), which comprises three components customized for the iris recognition context and datasets. IResNet does not need nor depend on data augmentation, making it a suitable core for the proposed IRIS-QResNet. Figure 3 illustrates the three components of IResNet: the stem block for handling input data, the residual stages, and the classification head, and shows the quanvolutional layer added to IResNet to extended to IRIS-QResNet.

#### 3.1.1. Stem Block for Input Handling

The stem block of IResNet converts the raw iris image into a compact and discriminative feature representation. It has been redesigned to work with a grayscale iris image *I*, with feature map *x*, as shown in Equation (1).

(1) $$x\epsilon R^{H\times W\times C_{in}}\;,\;\;\;C_{in}=1,\;$$

where R is the set of real numbers, H and W are the spatial dimensions, and $C_{in}$ denotes the grayscale. This stem block aims to suppress noise and illumination irregularities that are typical in iris datasets while performing early spatial abstraction, normalization, and nonlinear activation, thereby preserving low-level texture cues, such as furrows, crypts, and pigment variations. For each input iris image *I*, we use a compact and smaller 3 × 3 kernel for convolution $\left({Conv}_{3\times 3}\right)$ with two strides, Batch Normalization (BN), Swish activation (σ), followed by 3 × 3 Max-Pooling (MaxPool) instead of the original 7 × 7 kernel of the convolutional layer found in standard ResNet-18. In addition to preserving computational efficiency, this modification lessens over-smoothing and enhances the preservation of micro-level iris structures illustrated in Equation (2):

(2) $$f_{stem}(x)=MaxPool(\sigma (BN({Conv}_{3\times 3}(I)))\;,$$

The Swish activation function is defined as follows in Equation (3):

(3) σ(x)=x·sigmoid(x) ,

and is preferred over ReLU for small and medium-sized datasets due to its smoother gradient flow and more reliable empirical convergence [55]. Swish (also known as SiLU, or Sigmoid-weighted Linear Unit) provides distinct advantages through improved gradient propagation. Unlike ReLU, which outputs zero for all negative inputs, Swish retains a non-zero derivative even for negative values where Equation (4) expresses it as

(4) $$\sigma^{\prime }\left(x\right)=\sigma \left(x\right)+x\cdot \sigma (x)\cdot (1-\sigma \left(x)\right)\;,$$

allowing gradients to propagate backward through the network and mitigating vanishing gradient issues in deeper architectures [56]. Swish’s smooth, non-monotonic activation characteristics allow it to suppress large negative responses while selectively retaining small negative activations that may encode subtle discriminative cues [57]. This property is particularly advantageous in iris recognition, where fine-grained structures, such as crypts, furrows, and collarette variations, are often represented by low-magnitude activations that must be preserved to maintain discriminative power under limited-sample conditions. This lighter stem design preserves fine-grained iris texture details and lowers parameter count and overfitting risk in small datasets. Such capabilities are essential for precise recognition, particularly in datasets with few samples per class. By reducing the number of learnable parameters, the smaller kernel size also enhances model generalization in training scenarios with limited resources. The batch normalization momentum (β = 0.9) and a lightweight configurable *L*2 regularization term was empirically chosen to stabilize convergence without suppressing subtle discriminative features. The stem block produces a 64-channel normalized feature map with improved local contrast and decreased spatial resolution. As inputs to later residual stages, these representations offer the best possible trade-off between computational efficiency and spatial detail. Overall, the customized stem block offers a minimalistic yet expressive front-end, designed to preserve iris texture in cross-sensor and small-sample cases. Given its smaller kernel size, Swish activation, and precisely calibrated yet configurable regularization, it is a crucial component of the model’s robustness and generalization ability.

##### Kernel Standardization for Fair Comparison

While standard ResNet employs a 7 × 7 stem, the original IResNet configuration used heterogeneous kernel sizes distributed as 7, 3, 3, 3, 3, 1 across the stem and residual layers, with the quanvolutional layer using a 1 × 1 quantum measurement kernel. To ensure fair evaluation across multiple, diverse, and challenging iris datasets (e.g., MMU with eye-side information and UBIris) and to prevent dataset-specific biases, all convolutional kernels were standardized to 3 × 3 in both the customized baseline IResNet and the proposed IRIS-QResNet. Maintaining a larger 7 × 7 stem in the classical model would have granted a broader initial receptive field and introduced bias into performance comparisons, potentially inflating classical performance relative to the quantum-enhanced model. While some performance variations were observed with the original heterogeneous kernel distribution, the unified 3 × 3 configuration provides a consistent framework for evaluating the contribution of the quanvolutional layer. This choice was not introduced as an optimization strategy but to ensure receptive-field parity across architectures, thereby isolating the benefits of quantum-inspired enhancements rather than stem redesign.

#### 3.1.2. Residual Stages

**Figure 4:**
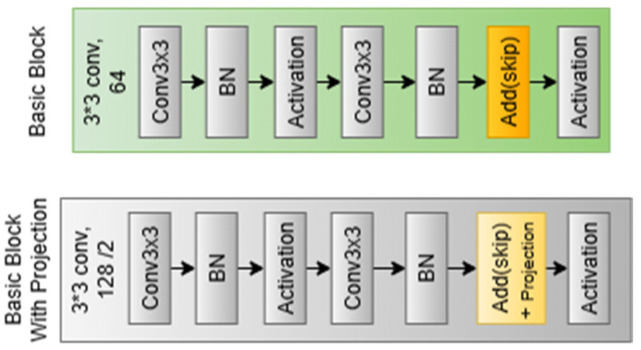
Building blocks of a single residual block, (**top**) the basic block, and (**down**) the basic block with the projection process.

The core of the baseline model is made up of residual stages, which allow for deep representation learning of iris textures and hierarchical feature extraction. The receptive field gradually widens during these phases, enabling the model to represent local and global iris features, such as cornea patterns, furrows, and crypts. Even in situations with limited training data, the residual design ensures stable convergence by maintaining gradient flow and reducing degradation in deeper networks. In line with an 18-layer depth, the baseline model consists of four residual stages with filter sizes [64,128,256,512] and repeat configurations of [2,2,2,2]. Each step consists of stacked basic residual blocks, as shown in Figure 4, with two 3 × 3 convolutional layers ${Conv}_{3\times 3}$ in each block. Batch normalization and Swish activation are then applied. One block can be mathematically expressed as follows in Equation (5).

(5) $$y=\sigma \left(BN\left({Conv}_{3\times 3}(x\right)\right)+F(x),$$

where F(x) is the residual connection that, when required, aligns dimensions using a 3 × 3 projection, x stands for the input tensor, or literally the features come from the last block, and y is the block output.

The residual stages of IResNet differ from those of general ResNet implementation in several ways. First, the benefit of using Swish activation rather than ReLU is that it produces smoother gradients, improving training stability and discrimination across minute changes in iris texture. Second, across different datasets, a lighter configurable L2 regularization and a high batch normalization momentum (m = 0.9) are added to reduce overfitting without compromising representational capabilities. Third, stride-2 convolutions are used for each downsampling operation between stages instead of pooling, which improves feature compactness for iris recognition and permits a more learnable spatial reduction. This combination provides a more favorable trade-off between robustness and model complexity.

**Figure 5:**
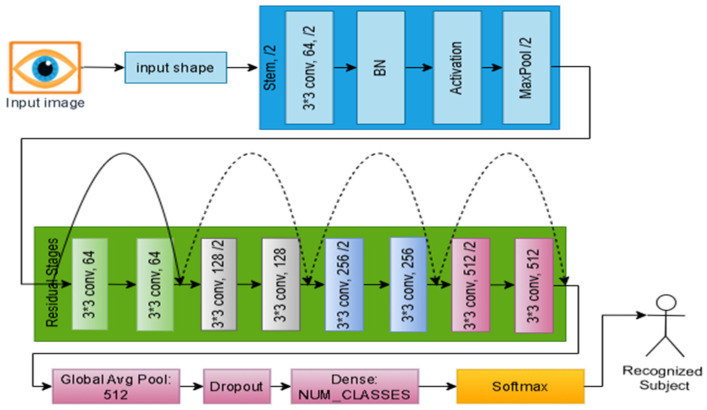
Detailed diagram of the IResNet baseline, solid arrows represents the normal connections, dashed arrows illustrates the skip connections.

While deeper stages (256 and 512 filters) capture more abstract global discriminative structures, the early residual stages (64 and 128 filters) concentrate on fine-grained localized patterns within the iris texture. Following the last phase, a high-level feature tensor is created, which contains the macrostructural and microtextural data required for accurate identity representation. These customized residual stages make up a balanced hierarchical encoder that maintains computational efficiency while preserving discriminative iris information. Swish activations, optimized normalization parameters, and learnable downsampling procedures are integrated to guarantee the network’s resilience in noisy cross-sensor and limited-sample acquisition situations. Figure 5 shows a detailed workflow for IResNet, where each residual block maintains a direct skip connection between its input and output. A projection shortcut employing a 3 × 3 convolution $\left({Conv}_{3\times 3}\right)$ and batch normalization aligns the dimensions when the input and output dimensions differ, ensuring correct residual addition. Without necessitating architectural changes, this mechanism maintains gradient flow across depth and stabilizes training.

#### 3.1.3. Regularization and Classification

This final part of the baseline IResNet architecture combines deep representations into discriminative identity judgments. It accomplishes two main goals: (1) forcing structural regularity on the learned features to improve generalization in the case of limited biometric data and (2) converting spatial feature maps into a condensed class-probability representation appropriate for iris identity prediction. The network introduces a controlled regularization step prior to classification, selectively disrupting intermediate activations during training. This encourages the model to use distributed representations instead of local features committed to memory. To prevent overfitting and improve the resilience of extracted iris embeddings against changes in illumination, noise, and occlusions, such regularization serves as a constraint that restricts co-adaptation between neurons. A global average pooling operation is used to aggregate the multi-dimensional feature maps following spatial encoding through the convolutional and residual hierarchies. This process efficiently converts the learned feature hierarchy into a condensed global descriptor of iris texture by compressing spatial dimensions while maintaining the most prominent activation responses. To guarantee that every sample is mapped to one of the predetermined identity classes, the pooled feature vector is subsequently run through a fully connected (dense) layer with a SoftMax activation function that produces normalized class probabilities, as follows Equation (6):

(6) $$\hat{y}=\frac{e^{z_{i}}}{\sum_{j=1}^{C}e^{z_{i}}},$$

where $\hat{y}$ represents the class probability distribution (i.e., SoftMax), *z_i_* is the final logit associated with the *i*th class, and C is the number of iris classes (identities). The classification and regularization procedures ensure that the model preserves a balance between generalization and discriminative precision. The SoftMax-based classification layer converts the high-level learned features into identity probabilities that can be understood semantically, while regularization enforces smoother parameter landscapes and reduces overconfidence. By connecting high-level decision formation with low-level feature extraction, this combination completes the architectural pipeline. As the last interpretive link in the baseline model, this stage essentially transforms learned spatial representations into categorical predictions, while preserving the model’s stability and generalization. It also guarantees the robustness and dependability of the decision-making process in iris recognition by combining feature regularization with global pooling and probabilistic mapping.

### 3.2. IRIS-QResNet: The Enhanced IResNet by a Quanvolutional Layer

Since iris textures naturally display intricate nonlinear patterns in both radial and angular directions, integrating such patterns for iris recognition is motivating. Quanvolutional representations employ mixing dynamics and trigonometric embeddings to enhance feature diversity without the need for artificial data augmentation, whereas CNNs frequently impose locality biases that may limit their capacity to capture subtle variations.

#### 3.2.1. Quantum State from Classic Input

Prior to the quantum encoding process, the preparation phase for the classical-to-quantum state, i.e., the normalization state, can be mathematically formulated by Equation (7) as:

(7) $$|\psi \rangle =\sum_{i=1}^{n_{qubits}}\alpha_{i}|i\rangle ,$$

In this case, |ψ⟩ is the general pure sate, |*i*⟩ indicates the qubits’ computational basis states (i.e., ∣0⟩, ∣1⟩, ∣2⟩, …, ∣ $n_{qubits}$ − 1⟩), $\alpha_{i}$ denotes complex amplitude coefficients obtained from the output features of the previous step, usually represented in Equation (8) as:

(8) $$\alpha_{i}=\alpha_{i}+\beta_{i}i\;,$$

which measures the ratio of the contribution of every computational basis state (*|i*⟩ to the total quantum states *|ψ*⟩), while *|ψ*⟩ or in Dirac notations “ket”, is the quantum state vector of a system consists of $n_{qubits}$, and the summation $\sum \begin{array}{c} n_{qubits}\\ i=1 \end{array}$ means the state *|ψ*⟩ is a superposition of $n_{qubits}$ basis states and runs over all the possible basis state for the identified value of $n_{qubits}$. This makes it possible for quantum computers to execute numerous computations at once. The amplitude coefficient $\alpha_{i}$ must also satisfy the condition of normalization expressed by Equation (9) as:

(9) $$\sum_{i=1}^{2^{n_{qubits}}}{\left|\alpha_{i}\right|}^{2}=1.$$

This equation ensures that the total probability of all measurement outcomes equals 1, where the square ${{|\alpha }_{i}|}^{2}$ is the possibility of measuring the basic state. With a probability denoted by ${{|\alpha }_{i}|}^{2}$, the system collapses to one of the basis states *|i*⟩. The essence of quantum states in superposition is encapsulated in Equation (9), which shows how quantum mechanics is probabilistic and how coefficients affect measurement results.

#### 3.2.2. The Proposed Quanvolutional Layer

**Figure 6:**
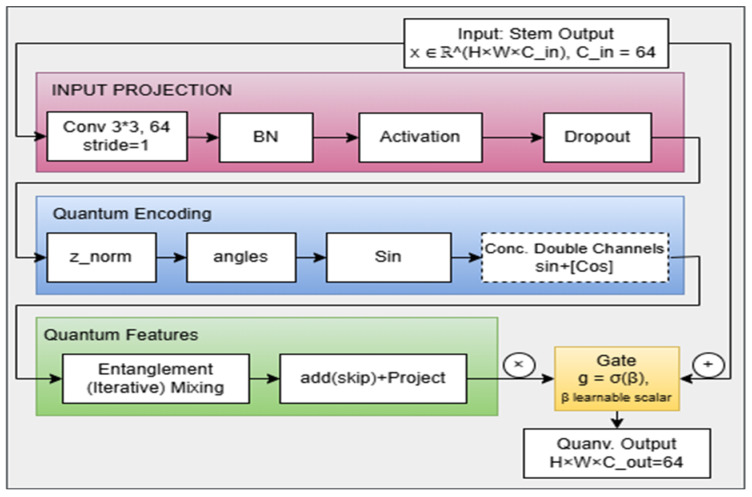
Detailed architecture of the proposed quanvolutional layer, shown as abstracted red box in Figure 3.

Figure 6 shows the detailed architecture of the quanvolutional layer that was abstracted and shown earlier as a red box in Figure 3. While alternative placements (e.g., before the stem or at multiple locations) are theoretically possible, this study specifically reports the results for the post-stem placement. The chosen position is motivated by conceptual and computational considerations, as described above, and provides a consistent framework for evaluating the impact of the quanvolutional layer. Systematic exploration of alternative placements will be considered in future work. The layer performs quantum-inspired feature encoding through sine–cosine transformations followed by convolutional mixing and gated residual integration. Its design ensures that enhanced features complement the baseline convolutional output without creating shape mismatches across residual paths. The layer starts by receiving an input feature map in a formal sense that represents the spatial dimensions and the number of channels, respectively (i.e., it is the exact output of the baseline stem block). This layer has a two-option pathway, depending on the required projection, to avoid output shape mismatch between the inner layers of the quanvolutional layer and the residual layers that receive the overall output from the quanvolutional layer.

##### Input Projection (Stem Transformation)

For the long path, where no projection is needed, the stem block of the quanvolutional layer consists of a 2D convolutional $\left({Conv}_{3\times 3}\right)$ layer that has a stride = 1; not 2 as the stem of the baseline. Then there is a batch normalization block, followed by an activation block (swish). The other difference from the baseline stem block is that here we have a dropout, not a max pooling block. As such, the stem block can be expressed in Equation (10) as:

(10) $$z=\sigma (BN(Conv_{3\times 3}(x))),$$

The dropout is implemented to enhance robust features, but it does not change the shape of the activation output. Thus, this block maintains spatial and channel dimensionality, ensuring that the output *z* has the same shape as the required quantum channel count.

##### Quantum Encoding (Feature Normalization and Sinusoidal Mapping): Classic-to-Quantum Conversion

Each feature in z is normalized using a bounded nonlinearity (tanh) to map the projected activations into a trigonometric embedding space inspired by quantum mechanics. This mechanism does not implement amplitude encoding, complex-valued rotations, or entangling gates. Instead, it provides a classical approximation of the rotational degrees of freedom common to single-qubit parameterizations, i.e., linearly scaled with structured trainable parameters: $\theta \in R^{n_{qubits}}$ and $\alpha \;\in R^{n_{qubits}}$. This normalization can be calculated using Equation (11):

(11) φ(x)=α⋅tanh(γz)+θ,

where z is the feature maps from the previous layer Equation (11), γ = 0.75 is a scaling constant (fixed in implementation), and the result of the equation is a set of angles φ(x) for trigonometric encoding. The trainable parameters are inspired by the angular update rules of qubit rotations but entirely implemented using real-valued classical operations. The tanh function ensures that feature values are constrained to the range [−1, 1], preventing unbounded phase values and improving training stability. The encoded angles are then transformed into trigonometric feature representations or the sine channel in Equation (12) is:

(12) $$q_{sin}\;=sin(\varphi (x))\;,$$

where the representation can be further enhanced by concatenating an optional cosine channel, expressed by Equation (13) as:

(13) $$q_{cos}\;=cos(\varphi (x)),$$

This dual-channel encoding follows the Euler identity ($e^{i\varphi }$) expressed in Equation (14) as

(14) $$e^{i\varphi }=\cos\left(\varphi \right)+i.sin(\varphi ),$$

enabling simulation of complex quantum amplitudes using real-valued tensors. The quantum feature tensor that results from the concatenation, in Equation (15), is:

(15) $$Q\left(x\right)=\;\{\begin{array}{c} q_{sin},\;\;\;\;\;\;\;\;\;\;\;\;\;\;\;\;\;\;\;\;\;\;\;\;\;\mathrm{if}\;\mathrm{cosine}\;\mathrm{is}\;\mathrm{not}\;\mathrm{used}\\ {[q}_{cos}||q_{sin}],\;\;\;\;\;\;\;\;\;\;\;\;\;\mathrm{if}\;\mathrm{cosine}\;\mathrm{is}\;\mathrm{used}\;\;\;\;\;\;\; \end{array}\;,$$

where || denotes the concatenation process. Using both channels doubles the dimensionality and produces a richer embedding that encodes phase-like geometry in feature space. Although no actual quantum states or complex amplitudes are formed, the paired trigonometric channels generate correlated variations that later layers can exploit in an “entanglement-like” manner.

The sine–cosine encoding is employed as a trigonometric transformation that maps classical feature activations into a form compatible with quantum-inspired operations. Prior work has shown that encoding input vectors with sine-based angle transformations introduces nonlinearity and periodicity, allowing the model to capture circular interactions among feature components that mimic convolutional behavior in quantum circuits [58]. This encoding also provides a structured representation suitable for quantum-inspired mixing layers, where the entanglement-like operations can exploit correlations across channels. Such transformations have been used successfully even with limited data, demonstrating robustness under finite-sample conditions and enabling feature extraction that is compatible with real quantum computations [59]. Positioning the sine–cosine encoding before the entanglement-like mixing step ensures that the resulting features retain these properties and can encode multi-channel dependencies in a manner analogous to quantum feature embeddings.

##### Quantum Entanglement Feature Mixing

Following the trigonometric encoding, the tensor *Q*(*x*) is processed according to Equation (16) by a sequence of convolutional mixing layers designed to capture cross-channel and spatial dependencies:

(16) $$M^{\left(d\right)}(Q)=\sigma (BN(Conv_{3\times 3}(M^{\left(d-1\right)}(Q)))),d\in \{1,\;\ldots ,\;D\}$$

where d is a depth parameter in the interval, and $M^{\left(d\right)}\left(Q\right)=Q(x)$ is the initial quantum feature tensor. Each iteration applies a 3 × 3 convolution, batch normalization, and a Swish activation. In all experiments, we set (D=2), This value balances computational efficiency and representational power. The complexity of the mixing stage scales is $O(H\times W\times C_{quantum}\times D)$, where *C_quantum_* is the quantum channel count (128 when combining sine and cosine encodings from 64 base channels). With (D=2), in all experiments, the process remains computationally efficient while providing two nonlinear mixing layers to recombine trigonometric encodings both spatially and across channels.

##### Entanglement-like Feature Mixing and Iris Texture Representation

Convolving sine–cosine channels produce emergent relational properties distinct from standard convolutional processing. Because these channels encode coupled angular embeddings, their joint transformation yields composite responses that depend simultaneously on local spatial context and the encoded angular phase. This creates cross-dependent relationships analogous to entanglement, without invoking quantum hardware or quantum operations. The mixed sine–cosine channel interactions create non-separable feature responses, enabling the network to encode joint variations in iris texture rather than isolated sinusoidal components. Through multi-scale relational mixing, small convolutional kernels unify micro-structures, crypts, furrows, and radial fibers, into spatially organized descriptors that better capture identity-bearing configuration patterns. Leveraging the unit-circle geometry of sine–cosine pairs, the model represents deformation phenomena such as curvature changes or rotational symmetries as stable angular-phase shifts, yielding implicit rotation-aware behavior. Together, these properties reorganize iris patterns into relational and geometric manifolds, enhancing discriminability beyond intensity-driven representations.

##### Significance for Iris Recognition

Iris individuality is defined by the relative arrangement of fine micro-structures. The quanvolutional layer captures these arrangements by encoding them into a geometric feature space in which spatial distortions, pupil dilation, and mild rotations are naturally accommodated. Rather than merely refining local features, the mixing stage reinterprets texture elements as components of a coherent geometric pattern, supporting more robust recognition under real-world variations. The mixing operation functions analogously to entanglement, as it couples the activation channels and enables distant or disjoint iris micro-patterns, such as crypts, furrows, and radial streaks, to be represented jointly rather than independently. This coupling generates feature vectors whose components encode non-local correlations, allowing the network to capture relationships between iris structures that classical convolutions, constrained by localized receptive fields, typically fail to model. As a result, the model learns integrated descriptors of iris texture that remain stable under dilation, rotation, and partial occlusion.

##### Output Projection and Gated Residual Integration

Following the mixing stage, the enhanced tensor $M^{\left(d\right)}\left(Q\right)$ is projected back to the original feature dimensionality using a 3 × 3 convolution followed by batch normalization in Equation (17):

(17) $$q_{out}=BN\left(Conv_{3\times 3}\left(M^{\left(D\right)}\left(Q\right)\right)\right).$$

This projected output is then fused with the residual pathway (the original input features) through a learnable gating mechanism that regulates the contribution of the enhanced features. Given g ∈ (0, 1), the final output of the quanvolutional layer is formulated by Equation (18) as:

(18) $$y=Res\left(x\right)+g\cdot q_{out},$$

where Res(x), the residual branch, When the input and output channel dimensions differ, Res(x) is implemented as a 3 × 3 convolutional projections followed by batch normalization to match dimensions as Equation (19) illustrates:

(19) $$Res\left(x\right)=BN\left(Conv_{3\times 3}\left(x\right)\right).$$

In cases of dimensional agreement, the residual reduces to identity mapping, expressed by Equation (20)

(20) Res(x)=x

##### Learnable Gate Parameter

The weighting coefficient, g ∈ (0, 1), is obtained or parameterized from a trainable scalar logit β, through a sigmoid activation in Equation (21) as:

(21) g=σ(β),

the parameter β = 0, to provide stability at initialization (g = 0.5) between the residual and enhanced pathways, and flexibility and adaptability during training as β is updated via backpropagation, allowing the model to learn how strongly the enhanced features should influence the final representation. A learnable gate adaptively fuses baseline and quantum-inspired pathways, preserving balanced gradient flow and preventing premature dominance of a single source. Empirical gate values remaining within the 0.3–0.7 range indicate consistent, moderate contribution from the quantum pathway, enhancing interpretability while ensuring that quantum-inspired features complement rather than override standard convolutional processing.

#### 3.2.3. From Quantum Formalism to Classical Implementation

The quantum state formalism presented in Equations (7)–(9) serves as a conceptual foundation for the design of our quanvolutional layer. While these equations describe the structure of true quantum states, characterized by complex amplitudes, phase rotations, and normalization constraints, the implementation used in our model is entirely classical. Accordingly, formalism is not executed on quantum hardware and does not involve complex-valued computation, unitary transformations, or amplitude encoding in the strict quantum-mechanical sense. Instead, the formal framework motivates a set of classical transformations designed to approximate certain representational properties of quantum systems while remaining fully compatible with convolutional neural networks. The trigonometric encoding uses Euler’s identity to map each angle parameter into paired cos(φ) and sin(φ) channels, forming a real-valued analog of complex amplitudes that fits naturally into standard CNNs. This two-channel structure provides a superposition-like embedding, where information is expressed through angular variation rather than intensity, yielding richer multicomponent features. A tanh-based normalization keeps angles bounded, stabilizing training by preventing divergence and maintaining consistent phase representations. The method remains strictly quantum-inspired, as all operations, real-valued activations, deterministic inference, and classical convolutions, are executed on conventional hardware. Although classical, the approach parallels prior quantum-inspired models by leveraging geometric properties of parameterized quantum states, and its sine–cosine channels, phase transformations, and cross-channel mixing serve as practical analogs of complex amplitudes, phase rotations, and entanglement-like correlations.

## 4. Experimental Setup

As we intend to compare the performance of both our models, the baseline IResNet and the enhanced IRIS-QResNet, we need to specify the experimental setups for evaluating their performance in identification and authentication.

### 4.1. Datasets and Preprocessing

We use pre-acquired well-known benchmark datasets to evaluate our proposed model, including CASIA-Iris-Thousand, IITD Database, MMU-Iris-Database v1, and UBIRIS_v1. The Institute of Automation, Chinese Academy of Sciences (CASIA), has offered a group of datasets. CASIA-Iris-Thousand [60] is a dataset that includes 20,000 images for 1000 subjects. In this research, we use only 5 images per eye (right/left) side and 10 per subject from the first 800 subject, namely a total of 8000 images. Kumar and Passi from the Indian Institute of Technology, Delhi (IITD) [61] collected iris images of 224 subjects,10 per subject, and 5 per eye side. Although this dataset has 2240 images, the first 13 people have images of only the left eye, resulting in only 2175 images for side-specific iris recognition. The Malaysian Multimedia University (MMU) [62] collected iris images from 45 subject, 10 per subject and 5 per right/left eye. The University of Beira (UB), Covilhã, Portugal, created the first version of the UBIris dataset [63] to obtain a noisy dataset with blurred, unfocused, and rotated images, which can also be closed and have light reflections besides the eyelids and eyelashes obstructions. A total of 1214 images of a single eye per Subject were acquired from 241 subject. We use only 1205 images from subjects who have 5 images.

**Figure 7:**
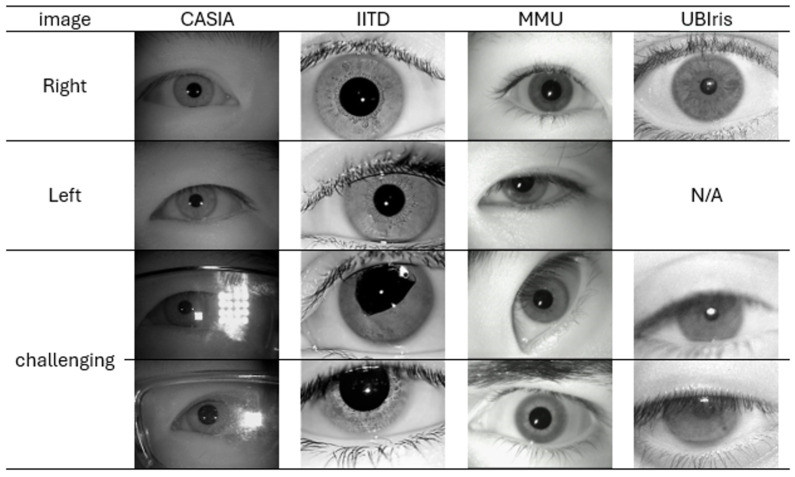
Examples of the four datasets samples, showing right, left and some samples of challenges.

Figure 7 presents examples of eye images from each of the four datasets. The table includes samples of both the right and left eye, and illustrates the distinct challenges associated with each dataset. In the CASIA dataset, reflections from glasses and variations in lighting conditions represent major issues. Additionally, the iris often appears smaller due to greater camera distance compared to other datasets. The IITD dataset includes images of eyes affected by surgeries or medical conditions, which can change iris overall shape and texture. The MMU dataset contains images where the eyes are not looking straight ahead and where different illumination wavelengths make the same iris appear as two concentric circles instead of one. UBIris has blurry eyes and fatigued eyes which can make the pupil appear distorted or illusory. These challenges typically necessitate preprocessing steps before further analysis to ensure fair model evaluation. Therefore, and for preprocessing, as the four datasets differ in size or color mode, we standardized all images to be in grayscale with OpenCV and resize to 160 × 120 to maintain consistent input dimensionality across datasets while preserving texture details. After that, we evaluate two experimental configurations to assess model robustness: End-to-End and Non-End-to-End approaches. In End-to-End approach, Raw 160 × 120 grayscale images (after resizing only, with no segmentation applied) are fed directly into the network. The model is therefore required to implicitly address occlusions, reflections, and iris localization through its convolutional layers. This setup serves to evaluate the model’s robustness under real-world, unconstrained imaging conditions. On the other hand, to apply the Non-End-to-End approach, pupil and iris boundaries were localized using coordinates stored in Excel files, obtained through a semi-automated process in which automated detection was applied first, and manual correction was performed only when required. Automated localization used median filtering (5 × 5) followed by Circular Hough Transform for pupil detection, and iris radii were estimated geometrically by adding dataset-calibrated constants in {15, 20, 25, 50, 75} to the pupil center. Results were validated by enforcing anatomical constraints (e.g., pupil radius is less than iris radius, then center difference is less than 10 pixels). Automated detection errors (8.4% CASIA, 2.5% IITD, 3.6% MMU, 6.5% UBIris) were corrected through a standardized interface in which overlays were displayed, operators verified accuracy, and incorrect circles were replaced by manual detection of the pupil center and one iris-boundary point; corrected coordinates were saved to Excel and used directly in all subsequent processing. Operators were blinded to subject identity, and verification was completed before train/test splitting to prevent bias. Using the verified coordinates, pupil and iris circles were drawn with OpenCV circle, and a binary mask was generated by filling the iris boundary in white and the pupil in black, retaining the iris annulus via OpenCV bitwise AND. Segmented regions were normalized using Daugman’s rubber sheet model with 80 radial and 320 angular samples, mapping each angle (θ) to Cartesian coordinates as expressed by Equation (22):

(22) $$\left(x,y\right)=((\cos\left(\theta \right)+x_{c}),(\sin\left(\theta \right)+y_{c})),$$

where $x_{c}$ and $y_{c}$ are the coordinates of the pupil, yielding 80 × 320 normalized iris images. Eyelid and eyelash occlusions were removed by processing each half of the normalized image using intensity inversion, thresholding (*T* = 0.9), morphological erosion, removal of small components (<10 pixels), and parabolic fitting to the upper occlusion boundary, with pixels above the fitted curve replaced by the mean iris intensity before recombining both halves. Optional enhancement variants included median filtering (5 × 5) and CLAHE (clipLimit = 5.0, tileGrid = 8 × 8) [64]. A summary of the resulting images per class and dataset is shown in Table 1, where there are seven groups of images that were used to train, validate, and then test the models. For testing both models in the Non-End-to-End and End-to-End approaches, using the seven dataset groups for each, which led to 14 main experiments outlined in Table 2. Every main experiment has two sub-experiments: one with the baseline and the other with the enhanced model. This results in 28 experiments.

### 4.2. Setting Parameters

As with any recognition model, we need to setup parameters to help the model perform its tasks as efficiently as possible. However, as we need to compare the performance of the baseline with the enhancement model, we need to carefully set these parameters to avoid bias, as they will have the same values for both the baseline and the enhanced model within the same experiment. In this subsection, we discuss two types of parameters:

#### 4.2.1. Dataset-Customizable Parameters

As listed in Table 3, the hyperparameters were adjusted according to the characteristics of each dataset to ensure stable and effective optimization while maintaining fair comparability between the baseline IResNet and the enhanced IRIS-QResNet. Although the overall optimization strategy remained unchanged, dataset size and complexity required modifying specific training parameters. Smaller datasets, such as CASIA, UBIris, and MMU, necessitated stronger regularization (higher L2 weight decay and dropout rates) to mitigate overfitting, whereas the moderate-sized IITD dataset enabled longer training with reduced regularization. The batch size was fixed at 16 for all datasets except IITD, where a larger batch size yielded smoother gradient updates. Learning rates were selected empirically per dataset CASIA/UBIris (2 × 10^−4^), IITD (3 × 10^−4^), and MMU (4 × 10^−4^), reflecting the inverse relationship between dataset size and the need for gradient exploration in small-sample regimes. A ReduceLROnPlateau scheduler (patience = 6, factor = 0.5) further stabilized convergence. To ensure a fair comparison, the baseline and quantum-enhanced models always used identical hyperparameters within each experiment. During development, certain challenging conditions, particularly the MMU End-to-End (eye-side) setting, required repeated stabilization. To avoid over-specializing hyperparameters to a single subset, a consistent selection criterion was adopted: if at least two of the four groups of a dataset achieved ≥90% accuracy under a particular configuration, that same configuration was applied to the remaining groups, even when their accuracies differed. This strategy preserved reproducibility and stability across heterogeneous datasets while preventing parameter tuning from artificially inflating the observed benefit of the quanvolutional layer.

L2 regularization was applied specifically to convolutional and dense kernels using the kernel_regularizer parameter in TensorFlow/Keras, while batch normalization parameters were excluded to avoid constraining scale and shift statistics. The regularization strength λ was dataset-dependent: $\lambda_{L2}=1\times 10^{-6}$ or small datasets (CASIA, UBIris) and $\lambda_{L2}=5\times 10^{-6}$. or medium-scale datasets (IITD), complemented by weight decay through AdamW ranging from 1 × 10^−6^ for small datasets to 5 × 10^−5^ for larger datasets. This decoupled implementation prevents redundant penalization during parameter updates and preserves discriminative iris textures essential for recognition performance. To ensure reproducibility, gallery and probe sets were constructed in a subject-disjoint manner. For identification experiments, a single reference image per eye per subject formed the gallery, while the remaining images comprised the probe set. Authentication experiments used genuine scores computed between corresponding probe and gallery images, while impostor scores were obtained by comparing probe images with non-matching gallery entries. Authentication thresholds were determined per dataset by optimizing the Equal Error Rate (EER) on a validation subset prior to testing. For IRIS-QResNet, classical feature maps extracted by the stem block were converted to quantum state coefficients via trigonometric encoding. Normalized pixel intensities were scaled into rotation angles for quantum gates, and parameterized quantum circuits were applied to small patches of these feature maps. Outputs were then mapped back to classical tensors through measurement expectation values, enabling seamless integration with subsequent convolutional layers while preserving gradient flow.

#### 4.2.2. Common Parameters

Common parameters are set with the same values for both models in all experiments:

- **Optimization:** Both models were trained using identical optimization settings to ensure strict comparability. The AdamW optimizer, which combines Adam’s adaptive moment estimation with decoupled weight decay, was used throughout all experiments. Parameter updates follow Equation (23):
  (23) $$\theta_{t\_1}=\theta_{t}+\frac{\eta \cdot \hat{m_{t}}}{\sqrt{\hat{v_{t}}}+\varepsilon }-\eta \lambda \theta_{t},$$
  where θ represents the trainable parameters vector (i.e., vector of weights and biases) of all trainable parameters at time *t*, η represents the learning rate that rescales the updates, $\hat{m_{t}}$ and $\hat{v_{t}}$ are bias-corrected first and second moment estimates, ε is a numerical stability constant, and λ is the weight decay coefficient. Using the same optimizer configuration isolates the effect of the quantum-inspired modules and ensures that both architectures are optimized under identical dynamics. During training, we monitored gradient norms, validation-loss evolution, and weight-update magnitudes for both IResNet and IRIS-QResNet. In all experiments, the optimizer produced smooth, monotonic decreases in validation loss without oscillations, divergence, or abrupt spikes. Importantly, the quantum-inspired blocks did not introduce additional gradient instability beyond what is typical for residual CNNs, confirming that the shared AdamW configuration yields stable and well-behaved convergence for both networks.
- **Regularization, weight decay, and loss function:** Regularization strategies were applied consistently across both architectures. Dropout was used as described in Equation (10), and total training loss was defined by Equation (24):
  (24) $$L_{total}=L_{\left\{CE\right\}}+\lambda \sum {\left||\theta |\right|}^{2},$$
  where $L_{total}$ is the total loss that the model aims to decrease, $L_{\left\{CE\right\}}\;$ is the sparse categorical cross-entropy loss, which can be computed in Equation (25) as follows:
  (25) $$L_{\left\{CE\right\}}=-\sum_{i=1}^{N}y_{i}log(\hat{y_{i}}),$$
  where *y_i_* is the true label, $\left(\hat{y_{i}}\right)$ is the predicted probability, and *N* is the number of classes. The term $\lambda \sum {\left||\theta |\right|}^{2}$ is a regularization element, particularly L2, also known as weight decay. Here, to prevent overfitting, the regularization strength, represented by λ, controls the trade-off between fitting the data and maintaining small model weights, promoting simpler models that perform better when applied to unseen data. *θ* is the parameters or weights of the model, and ${\left||\theta |\right|}^{2}$ represents the squared L2 norm of these parameters, where it can be simplified by Equation (26) as:
  (26) $${\left||\theta |\right|}^{2}=\sum_{j=1}^{N}\theta_{j}^{2},$$
  This formulation helps prevent overfitting by constraining parameter magnitudes, promoting better generalization to unseen iris samples.
- **Gradient flow optimization:** Residual connections play a central role in stabilizing gradient propagation, enabling both models to learn discriminative features without the need for heavy data augmentation. The gradient flow through each residual block is given by Equation (27):
  (27) $$\frac{\partial L}{\partial x_{l}}=\frac{\partial L}{\partial x_{l+1}}\;\cdot (1+\frac{\partial F}{\partial x_{l}}),$$
  where L stands for the loss gradient relative to the input $x_{l}$ of layer *l*, and $\frac{\partial L}{\partial x_{l}}\;$ is the gradient with respect to the input of layer *l*. The gradient propagated from the next layer, $\frac{\partial L}{\partial x_{l+1}}$, in relation to the output $x_{l+1}$, and $\frac{\partial F}{\partial x_{l}}$, is the derivative of the residual function *F* (typically the convolutional output excluding the skip connection) with respect to $x_{l}$. It illustrates how variations in the input to the residual blocks affect their outputs. The statement $1+\frac{\partial F}{\partial x_{l}}$ signifies the effect of the identity mapping and the residual function. The identity mapping. The value 1 denotes that the gradient flow via the skip connection is still intact, while $\frac{\partial F}{\partial x_{l}}$ reflects the extra gradient contribution brought about by transformations of the residual functions. The skip connection guarantees that gradients remain at least as large as those propagated from the next layer, even when the residual branch contributes only a small derivative. This mechanism is especially important for IRIS-QResNet, where the quantum-inspired gated pathway introduces additional nonlinear transformations. The preserved identity component ensures that vanishing gradients cannot occur even when gating attenuates the residual branch, allowing AdamW (Equation (23)) to maintain stable updates throughout both architectures.
- **Feature space regularization**: Implicit regularization is further supported by batch normalization and dropout, which reduce sensitivity to variations in iris appearance. The architecture itself provides multi-resolution analysis through progressive downsampling and channel expansion, enabling effective feature extraction without dependence on complex or artificial augmentation schemes. ResNet-18 remains one of the most reliable baselines for iris recognition due to its proven balance of representational strength and computational efficiency.
- A type of **multi-resolution analysis** is naturally implemented through architectural progression, where features are extracted at various spatial resolutions using channel expansion and progressive downsampling. ResNet-18 is among the best baseline architectures for iris recognition due to its theoretical underpinnings and empirical benefits. It offers strong feature extraction capabilities and computational efficiency without the need for intricate data augmentations.

To confirm that the presence of gated quantum-inspired residual paths did not alter optimization behavior, we explicitly measured convergence characteristics across all training runs. While AdamW can theoretically behave differently when encountering nonlinear gates and weight-sampling noise, both IResNet and IRIS-QResNet demonstrated stable and monotonic loss reduction. Layer-wise L2 gradient norms consistently fell within the range (0.3–1.7), and weight-update magnitudes remained stable across epochs. No oscillatory behavior, sudden spikes, or divergence, phenomena sometimes associated with gated residual networks, were observed. Representative convergence curves and gradient-norm trajectories are provided in next section to document optimizer stability and ensure full reproducibility.

All convolutional kernels, across the stem, residual blocks, and post-quanvolutional layers, were fixed to 3 × 3 to ensure fair architectural comparison. The quanvolutional layer was inserted immediately after the stem, where the input is reduced to ~40 × 30 or 80 × 20 while retaining key iris structures such as edges, gradients, crypts, furrows, and collarette patterns. Introducing the quantum-inspired transformation at this stage enriches these foundational descriptors before they propagate through deeper residual blocks. This early placement also ensures computational efficiency: spatial dimensions are already reduced by ~75%, and the channel depth remains manageable (64 channels), preventing the exponential cost that would arise in later layers. The computational complexity follows $O(H\times W\times C_{in}\times n_{qubits}\times D)$. Positioning the layer early provides stable gradient flow to all quantum parameters (θ,α,β), supported by AdamW, residual connections, L2 regularization, and dropout, yielding reliable convergence on CASIA, UBIris, IITD, and MMU without divergence. Since iris identity is encoded in fine-grained multiscale textures, quantum enhancement at the low-to-intermediate feature level offers the greatest discriminative gain, while deeper layers contribute diminishing returns. For small datasets (5–10 images per class), a single post-stem quantum layer increases representational richness without significant parameter growth, avoiding the overfitting risk associated with stacking multiple quantum layers.

### 4.3. End-to-End and Non-End-to-End Setups

**Figure 8:**
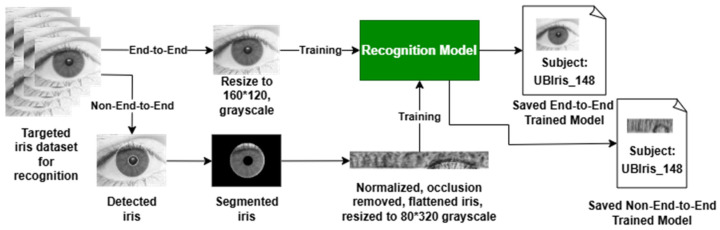
IRIS-QResNet using End-to-End and Non-End-to-End approaches for iris recognition.

The main purpose of the proposed IRIS-QResNet is to enable enhanced quantum-inspired capabilities in an End-to-End or a Non-End-to-End mode for iris recognition, as shown in Figure 8. As such, the model is able to handle images in two different sizes. First, we read the full eye image for the End-to-End approach, where the only preprocessing here is converting the image to grayscale. Moreover, as the datasets vary in image dimensions, we force all images to have a width (W) of 160 and a height (H) of 120. No further preprocessing is required as the model should select the most important pixels on its own. On the other hand, for the Non-End-to-End approach, we need to apply comprehensive preprocessing so that only the cropped iris with the fewest occlusions and anomalies is used for training the model. Thus, the preprocessing includes iris localization to select the iris area of interest, then segment it, normalize it to represent a pattern rectangle, remove all occlusions and anomalies as much as possible, and finally resize it to W: 320 and H: 80. In both approaches, the data are sent to train the model. As soon as training is completed successfully, the model is saved for further usage in biometric recognition tasks. Furthermore, since our datasets contain entirely distinct subjects, cross-dataset evaluation on the same identities is not feasible. To ensure rigorous testing, we divided each dataset into training, validation, and testing subsets, guaranteeing that at least one image per subject was excluded from training. The model was trained using *model.fit* on the training set, validated during training on the validation set, and evaluated on the held-out testing set. The reported accuracies therefore reflect performance on completely unseen subjects, demonstrating the model’s ability to generalize to new individuals within the same dataset. We also did not perform cross-evaluation between End-to-End and Non-End-to-End configurations within the same dataset (e.g., testing Non-End-to-End models on End-to-End images and vice versa) because the differing image dimensions would cause implementation errors. Therefore, evaluating the models on unseen data within the same experimental setup was the most appropriate and feasible approach for this study.

**Figure 9:**
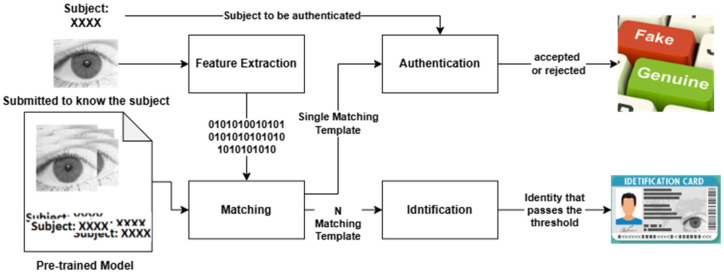
Authentication scenario vs. identification scenario using IResNet and IRIS-QResNet.

Identification and authentication are the two well-known scenarios for iris recognition. According to Jaha [65] identification is a one-to-many recognition problem, where we need to identify a subject using an unknown iris sample as an unseen query biometric template. In response to the query, the system ought to determine if this biometric template matches a known subject, and if so, the system retrieves the top-match identity from the enrolled individuals in the gallery. On the other hand, authentication is a one-to-one recognition problem, where a gallery is probed using an unseen iris sample to verify a claimed identity as a query biometric template. Based on the authenticity of the biometric template compared to the previously enrolled templates for that claimed subject in the gallery, the system should evaluate the query and determine whether it belongs to the claimed identity before accepting or rejecting it, in accordance with the confidence level control [65]. Figure 9 describes and differentiates how IResNet and IRIS-QResNet are used and evaluated in authentication and identification scenarios.

The main difference between the two scenarios is that in identification, we just match the iris to know whose it is, while in authentication, we match the iris against potential subject information to determine whether they are or are not the authentic subject. To this extent, in identification, as shown in Figure 9, the eye image, as if in the End-to-End approach, will be sent for feature extraction that will be matched with the pre-recorded data in the pre-trained model. The matching process enforces N-matching template, and then the result will be the subject data that passes the matching phase. In authentication, on the other hand, the model needs to obtain the potential subject data along with the eye image, so that the authentication process will match the single query template with the available subject data to be authenticated. The result here will just specify either to accept the subject claim as a genuine user or reject it as an imposter user.

### 4.4. Evaluation Metrics

Initially, to emphasize the novelty, superiority, and contributions of our research work compared to related literature, we introduce a comparative analysis and discussion, highlighting research gaps and aspects that this work addresses, while existing related studies either partially or never considered. Given the evident dissimilarities and absence of fairly comparable aspects between this work and existing work, quantitative comparisons are deemed unrepresentative and impractical. However, to overcome this challenge, we use the baseline model (IResNet) as a representative of classical models to compare the accuracy and loss of the proposed enhanced quantum-inspired model (IRIS-QResNet), with both models using the same values of the feeding parameters to ensure fairness and credibility across comparisons.

We evaluate our models using a range of standard biometric performance metrics. The Cumulative Match Characteristic (CMC) curve, used usually in identification scenarios, provides a cumulative measure of the models’ performance in correctly matching a probe image to its corresponding identity within a ranked gallery. The Rank-1 and Rank-5 recognition rates are major CMC performance indicators, respectively, indicating the likelihood of correctly identifying a person at the top match and among the top five ranked candidates. Moreover, we use the Area Under the CMC Curve (CMC-AUC) to summarize the overall identification accuracy across all ranks.

For authentication experiments, match score distributions were explicitly computed to evaluate genuine–impostor separability. For each subject, one template was treated as a query and matched against all enrolled templates. Scores from the same identity formed the genuine distribution, whereas scores from all other identities formed the impostor distribution. These distributions were used to compute the ROC curve, AUC, and Equal Error Rate (EER). The decision threshold was obtained at the intersection of the False Acceptance Rate (FAR) and False Rejection Rate (FRR). All authentication plots (genuine vs. impostor histograms, ROC curves) are included in next section as an example of one of the 14 experiments with the smallest but the hardest dataset, i.e., MMU End-to-End with Eye side. In addition, we examine the trade-off between the True Acceptance Rate (TAR) and the False Acceptance Rate (FAR) over different decision thresholds using the Receiver Operating Characteristic (ROC) curve. To evaluate authentication performance, we report the Area Under the ROC Curve (ROC-AUC), the Equal Error Rate (EER), and the decidability index (d′) While ROC-AUC quantifies the global discriminability of the system across all thresholds, the EER provides a threshold-specific operating point at which the False Acceptance Rate (FAR) equals the False Rejection Rate (FRR). In addition, the decidability index (d′) was computed to quantify the statistical separation between genuine and impostor score distributions, offering a direct assessment of biometric reliability and the robustness of score space separability. Because d′ is highly informative for evaluating the stability of the recognition system and is widely recommended in biometric performance reporting, numerical $d^{\prime }$ values for every dataset and each experimental configuration are reported in the Results section together with the score distribution plots. Performance evaluation of MMU End-to-End iris recognition with eye-side using IResNet and IRIS-QResNet. (a–c) show training accuracy and loss dynamics, (d) depicts genuine–imposter score distributions, (e–g) show verification and identification curves (DET, ROC, CMC), and (h–k) illustrate threshold-based performance metrics. IRIS-QResNet demonstrates improved separability and earlier convergence, confirming enhanced discriminative capacity under challenging small-sample conditions These complementary metrics collectively provide a complete assessment of both identification and authentication performance in accordance with contemporary biometric evaluation guidelines.

## 5. Experiment Results and Discussion

This section discusses the two models, IRIS-QResNet and its baseline IResNet, which are evaluated and thoroughly compared in iris identification and authentication. Our experimental design emphasizes controlled comparison to isolate the specific contributions of quantum-inspired architectural components while ensuring scientific rigor and reproducibility.

### 5.1. Comparison with the State of the Art

The quantum-inspired functionalities used in this research are considered transformation functions as they depend on the Quantum Fast Fourier Transformer (QFFT) that inherently modify feature representations. Thus, the proposed model (IRIS-QResNet) and its baseline (IResNet) for iris recognition do not use augmentation intentionally to ensure fair comparison, allowing us to isolate the contribution of the quantum-inspired transformations. However, this makes it harder to compare them with available solutions, as augmentation has always been used to increase the accuracy of deep models. Table 4 summarizes where our research differs, situates our work relative to recent handcrafted, deep, and quantum-inspired approaches. and shows how it will not be fair or comparably representative in quantitative terms with available solutions.

In handcrafted approaches, it is not compulsory to use augmentation if the model can learn from small datasets; examples are LBP with Haar wavelet models [14], Multiple methods fusion [66], and Hybrid BSIF + Gabor [67]. However, none of these models use a quantum-inspired approach or deep learning, although these are modern articles. For a deep CNN-based approach, DNN in general forces augmentation; if not, then it will be another type of transformer, such as wavelength transformer, python built-in image transformer, or QFFT, which is the basis of many quanvolutional layers. However, some researchers did not state it clearly, such as in the CNN model of Sallam et al. [68] and the deep SSLDMNet-Iris [25] models, especially Sallam’s suggested model, which did not specify whether it used augmentation or how many layers it has. Both models, in addition to Gabor with DBN [69], EnhanceDeepIris [70], and Multibiometric System [71] are not quantum-inspired, but the latter three clearly depend on data augmentation. Quantum-inspired articles do not depend on augmentation. QFFT or other quantum filters, their work directly contradicts with the data augmentation or other transformers, which lead to lose the features rather than increasing them or maximizing the data for rich features. This explains clearly why we forced no augmentation in this research. Examples from the state of the art: QiNN [72] using CIFAR-0 to enhance augmentation itself, QNN [50] using MNIST, and QCNN [48] using the iris flower dataset. QNN and QCNN do not use augmentation as the quanvolutional layer performs the transformation task to enhance the results. Some researchers tried to use iris recognition as the domain for their quantum-inspired works, such NAS-QDCNN [73] which reported accuracy raised with 96.83% without telling which dataset. There are also quantum algorithms [74], and Enhanced Iris Biometrics [75] which did not report any accuracy. Post-quantum authentication [76] used quantum inspired fundamentals to decrease the success possibility of any attack attempt.

Although the accuracies reported by our models may appear moderate compared to some dataset-specific works claiming near-perfect results, those studies typically depend on handcrafted features, augmentation pipelines, or dataset-tuned designs. IRIS-QResNet, by contrast, operates on large feature spaces (Table 5) without augmentation or hybrid handcrafted preprocessing. Although the accuracies reported by our models may appear moderate compared to some dataset-specific works claiming near-perfect results, those studies typically depend on handcrafted features, augmentation pipelines, or dataset-tuned designs. IRIS-QResNet, by contrast, operates on large feature spaces (Table 5).

**Table 4:** Summary of comparative gap analysis where our proposed work overpasses earlier work.

Approach	Technique Used	Biometric	Iris	Shared Dataset	Used Dataset	Segmented	Augmented *	Other Preprocessing	Quantum-Inspired	Deep Learning	Eye-Side Recognition	Achieved Accuracy	Reference
Handcrafted	LBP with Haar wavelet	✓	✓	✓: MMUv1	1. MMU1 2. MMU2	✘	✘	✓	✘	✘	✘	DCT_LBP_KNN: 85.71% DCT_LBP_SVM: 91.73% HWT_LBP_RF: 83.46%	[14]
	Multiple methods fusion	✓	✓	✓: CASIA	CASIA v4.0	✓	✘	✓	✘	✘	✓: Separate	Left eyes: 98.67% Right eyes: 96.66%	[66]
	Hybrid BSIF + Gabor:	✓	✓	✓: IITD	IITD	✓: partially	✘	✓	✘	✘	✘	95.4%	[67]
Deep CNN-Based	CNN	✓	✓	✓: CASIA	1. CASIA-Iris-V1 2. ATVS-FIr DB	✓ ✘	Unknown	Unknown	✘	✓	✘	Cropped IRIS:97.82% IRIS Region:98%	[68]
	deep SSLDMNet-Iris with Fisher’s Linear Discriminant (FLD)	✓	✓	✓: all	CASIA 1.0, 2.0, 3.0, 4.0, IITD, UBIris, MMU	Unknown	Unknown	✓	✘	✓	✘	CASIA: 99.5% IITD: 99.90% MMU: 100% UBIris: 99.97%	[25]
	Gabor with DBN	✓	✓	✓: CASIA	CASIA-4-interval, CASIA-4-lamp, JLUBR-IRIS	✓	✓	✓	✘	✓	✘	CASIA-4-interval: 99.998 CASIA-4-lamp: 99.904	[69]
	EnhanceDeepIris	✓	✓	✓: CASIA	ND-IRIS-0405, CASIA-Lamp	✓	✓	✓	✘	✓	✘	CASIA-Lamp: 98.88%	[70]
	Multibiometric System	✓	✓	✓: CASIA, IITD	CASIA-V3, IITD	✓	✓	✓	✘	✓	✓: Separate	IITD left: 99% IITD right: 99% CASIA left: 94% CASIA right: 93%	[71]
Quantum-inspired	QiNN	✘	✘	✘	-	-	✘	-	✓	✓	✘	-	[72]
	QNN	✘	✘	✘	-	-	✘	-	✓	✓	✘	-	[50]
	QCNN: iris the flower	✘	✓	✘	-	-	✘	-	✓	✓	✘	-	[48]
	Quantum Algorithms	✓	✓	✓: CASIA	CASIA V1.0	Not specified	✘	✓	✓	✘	Not specified	No recorded accuracy	[74]
	Enhanced Iris Biometrics	✓	✓	✓: UBIris	UBIris	Not specified	✘	✓	✓	✘	Single side	No recorded accuracy	[75]
	Post-quantum authentication	✓	✓	✓: UBIris	UBIris, another dataset	✓	✘	✓	✓	✘	Single side	No recorded accuracy	[76]
Our approach	IResNet (Baseline)	✓	✓	CASIA thousand, IITD, UBIris, MMU		✘	✘	✘	✘	✓	✘	CASIA: 97.50% IITD: 97.32% MMU: 77.78% UBIris: 94.61%	[This research]
											✓: +	CASIA: 71.19% IITD: 95.40% MMU: 50.00%	
						✓		✓			✘	CASIA: 90.63% IITD: 98.66% MMU: 93.33% UBIris: 89.21%	
											✓: +	CASIA: 78.50% IITD: 98.16% MMU: 77.78%	
	IRIS_QResNet (quantum-inspired)	✓	✓			✘	✘	✘	✘	✓	✘	CASIA: 97.88% IITD: 98.66% MMU: 86.67% UBIris: 97.51%	
											✓: +	CASIA: 71.38% IITD: 97.24% MMU: 66.67%	
						✓		✓			✘	CASIA: 92.25% IITD: 99.55% MMU: 97.78% UBIris: 95.02%	
											✓: +	CASIA: 80.56% IITD: 98.39% MMU: 88.89%	

* We aim not to use data augmentation to avoid contraction of the transformation. + Our models recognize by subject, e.g., Subject 20 and Subject 25, and by side-specific eye, e.g., the right eye of Subject 20 and the left eye of Subject 25, while not separating them into two datasets.

**Table 5:** Test results of IResNet and IRIS-QResNet, while varying datasets, approaches, and tasks.

Dataset	Metric	End-to-End (E2E)				Non-End-to-End (~E2E)			
		IResNet	IRIS-QResNet	Imp. %	with Eye Side	IResNet	IRIS-QResNet	Imp. %	with Eye Side
CASIA	Acc.	97.50	97.87	0.3750	✘	90.63	92.25	1.6250	✘
	Loss	0.11	0.07	−0.036		0.45	0.34	−0.1070	
	Parameters	12,954,336	13,139,297	185,473		12,954,336	13,139,297	185,473	
	CI	[0.9627, 0.9678]	[0.9718, 0.9763]	-		[0.8603, 0.87]	[0.8921, 0.9009]	-	
	g	-	0.4495	-		-	0.4252	-	
	Acc.	71.19	71.38	0.1870	✓	78.50	80.56	2.0630	✓
	Loss	1.94	1.81	−0.1220		1.17	1.01	−0.1550	
	Parameters	69,072,896	69,257,857	185,473		13,364,736	13,549,697	185,473	
	CI	[0.8258, 0.8365]	[0.7968, 0.8075]	-		[0.6665, 0.681]	[0.7372, 0.7507]	-	
	g	-	0.4621	-		-	0.4325	-	
IITD	Acc.	97.32	98.66	1.3400	✘	98.66	99.55	0.8930	✘
	Loss	0.25	0.12	−0.1380		0.14	0.06	−0.0790	
	Parameters	12,658,848	12,843,809	185,473		12,658,848	12,843,809	185,473	
	CI	[0.8798, 0.8978]	[0.9623, 0.9722]	-		[0.9253, 0.9382]	[0.9782, 0.9863]	-	
	g	-	0.4842	-		-	0.4833	-	
	Acc.	95.40	97.24	1.8390	✓	98.16	98.39	0.2300	✓
	Loss	0.41	0.22	−0.1930		0.55	0.17	−0.3790	
	Parameters	12,767,091	12,952,052	185,473		12,767,091	12,952,052	185,473	
	CI	[0.8196, 0.8426]	[0.9193, 0.9354]	-		[0.7016, 0.7244]	[0.9299, 0.9428]	-	
	g	-	0.4842	-		-	0.4848	-	
MMU	Acc.	77.77	86.67	8.8890	✘	93.33	97.78	4.4450	✘
	Loss	1.29	0.86	−0.4240		0.60	0.47	−0.1310	
	Parameters	12,567,021	12,751,982	185,473		12,567,021	12,751,982	185,473	
	CI	[0.7046, 0.754]	[0.8344, 0.8734]	-		[0.7734, 0.8133]	[0.932, 0.9556]	-	
	g	-	0.4904	-		-	0.4892	-	
	Acc.	50.00	66.67	16.67	✓	77.78	88.89	11.111	✓
	Loss	2.064	1.543	−0.6490		1.76	0.95	−0.8060	
	Parameters	12,590,106	12,775,067	185,473		12,590,106	12,775,067	185,473	
	CI	[0.3976, 0.4455]	[0.5595, 0.6115]	-		[0.4052, 0.4591]	[0.7473, 0.799]	-	
	g	-	0.4912	-		-	0.4910	-	
UBIris	Acc.	94.60	97.51	2.9040	✘	89.21	95.02	5.8090	✘
	Loss	0.41	0.16	−0.2530		1.44	0.42	−1.0280	
	Parameters	12,667,569	12,862,642	185,473		12,667,569	12,852,530	185,473	
	CI	[0.7849, 0.8153]	[0.9328, 0.952]			[0.3939, 0.4294]	[0.7994, 0.8291]		
	g	-	0.4870	-		-	0.4875	-	

Without augmentation or hybrid handcrafted preprocessing. In addition, cross-dataset evaluations show that IRIS-QResNet maintains high performance even under varying imaging conditions, a property often difficult to achieve without augmentation. t Its quantum-inspired behavior is hypothesized to arise from (1) sinusoidal encoding that maps spatial features into frequency-sensitive representations, (2) entanglement-like mixing that induces informative inter-channel dependencies, and (3) quantum-parallelism principles that support multi-scale feature extraction, which is particularly advantageous in small-sample biometric conditions. These features make IRIS-QResNet suitable for any dataset, regardless of its size or image resolution. The model can operate in two modes: End-to-End and Non-End-to-End. This flexibility allows us to select the dataset size to achieve optimal performance without requiring additional modifications to the model or designing dataset-specific models that may achieve perfect results on one dataset but perform poorly on others. Most iris-biometric solutions also do not account for eye side. Only a few works consider left/right recognition [53,66,71] and they train separate models for each eye. In contrast, our models are trained jointly on both eyes without side-specific bias, enabling a generalizable solution suitable even when only one eye is available.

For these reasons, direct quantitative comparison with state-of-the-art models would be inequitable. Classical pipelines (e.g., Gabor encoders, LBP-SVM) do not operate under identical training conditions, and recent studies emphasize comparing new architectures under unified preprocessing and optimization settings. Our controlled design isolates the contribution of quantum-inspired layers without interference from augmentation or cross-model variability. Future work will extend comparisons to classical and hybrid pipelines under matched augmentation conditions.

### 5.2. Test Accuracy and Loss

The enhanced IRIS-QResNet model was tested to compare its performance against that of its baseline IResNet counterpart. As we tried our best to unify the parameter values, at least while testing both models with the same dataset, we also performed tests with completely unseen data. Both models were evaluated on four widely used iris datasets, CASIA, IITD, MMU, and UBIris, across four recognition scenarios: (1) End-to-End subject recognition, (2) End-to-End with eye-side specification, (3) Non-End-to-End subject recognition, and (4) Non-End-to-End with eye-side specification. In standard recognition, all 10 images per class were used; when eye-side was required, only 5 samples per side were available. Table 5 reports the accuracy and loss values for all configurations.

Table 5 compares the iris recognition test results of the baseline and enhanced models (IResNet and IRIS-QResNet) in terms of accuracy and loss, while varying datasets, approaches, and tasks. IRIS-QResNet has higher accuracy and lower loss than IResNet across all test cases, confirming significant improvement and providing a better solution even for low-sample datasets. However, IITD yields the highest accuracy and lowest loss in all test cases for both models. The observed accuracy improvements amply demonstrate the generalization benefits of the quanvolutional layer. Even with very few training samples, the enhanced model can more effectively distinguish confusable iris classes by taking advantage of the suggested hybrid architectures, sinusoidal encoding, and entanglement-like feature mixing, which capture richer more discriminative representations than the baseline. IRIS-QResNet’s capacity to generalize to unseen iris images without augmentation supports the idea that quantum-inspired operations serve as an efficient regularizer.

On all four datasets (CASIA, IITD, MMU, and UBIris), Table 5 shows that IRIS-QResNet outperforms IResNet for both iris subject recognition approaches (End-to-End and Non-End-to-End). Interestingly, IRIS-QResNet continuously attains lower loss and higher accuracy, demonstrating superior feature extraction efficiency and generalization. For example, on CASIA, accuracy increases from 97.5% to 97.875% for End-to-End recognition and from 90.625% to 92.25% for Non-End-to-End recognition. At the same time, loss values decrease from 0.106 to 0.070 and from 0.447 to 0.340, respectively. This pattern repeats for IITD, where the robustness of the models on comparatively high-quality data is demonstrated by the accuracy peaking at 99.554% for Non-End-to-End recognition and the loss minimizing to 0.057. However, for End-to-End recognition, the accuracy increases from 97.32% to 98.66% and the loss decreases from 0.255 to 0.117. MMU exhibits the greatest relative gains, with smaller size and greater variability, resulting in accuracy jumps of over 10% and 4.5% for End-to-End and Non-End-to-End recognition, respectively. Significant reductions in loss demonstrate that IRIS-QResNet can effectively manage noise and data scarcity through its quantum-inspired modifications. On UBIris, IRIS-QResNet achieves 95.021% and 97.51% accuracy with significantly lower loss of 0.415 and 0.157 in Non-End-to-End and End-to-End recognition, respectively. Although performance decreases when the eye-side is specified compared with recognition without eye-side specification, IRIS-QResNet consistently outperforms the baseline IResNet under both settings. The results also show that, when the eye-side is specified, the Non-End-to-End protocol yields better performance than the End-to-End approach across all datasets. Table 5 further reports the learned values of the sigmoid gate g, confirming that the gating mechanism operated as intended, with values remaining within the range 0.3–0.7 and avoiding collapse. Confidence intervals (CI) are also provided to demonstrate statistical stability. These intervals provide a measure of reliability even for classes with very small sample sizes. While significance testing across all metrics was not included, the reported confidence intervals indicate that the observed performance gains from incorporating the quantum layer are consistent and not incidental. This demonstrates that the improvements are robust across datasets, modes, and evaluation splits.

The decrease in performance after incorporating eye-side in MMU dataset information is likely due to feature redundancy, as the model may extract overlapping information from both eyes, which can confuse rather than enhance classification. Additionally, dataset variability plays a role, since some datasets contain more consistent inter-eye patterns than others, making the eye-side signal less reliable. Finally, mode and complexity interactions (End-to-End vs. Non-End-to-End) can amplify subtle inconsistencies, causing a minor drop in accuracy despite overall robust performance across metrics. For recognition without specifying the eye-side, no single preprocessing strategy dominates: CASIA and UBIris perform better under the End-to-End protocol, whereas IITD and MMU benefit more from Non-End-to-End processing. Despite these differences, the overall findings support the claim that quanvolutional structures can enhance discriminative power even in small-scale iris datasets. The IRIS-QResNet architecture improves representation learning by leveraging quantum-inspired parallel mixing and non-local feature coupling, leading to more stable convergence [75] and stronger class separability. As a result, IRIS-QResNet achieves superior accuracy and maintains computational efficiency, representing a meaningful advancement for biometric recognition in limited-data regimes.

Importantly, IRIS-QResNet was consistently compared with IResNet using matched hyperparameters, and its superior performance is expected given its additional quanvolutional layer and approximately 185,473 extra parameters (Table 5). Parameter variations across groups of the same dataset arise from differences in the retained visual region after preprocessing; for example, the CASIA End-to-End (eye-side) setting includes surrounding structures such as skin, eyelashes, and eyelids, leading to over 69 million parameters due to expanded feature dimensionality. This standardized evaluation protocol ensures a fair and reproducible comparison between the classical and quantum-enhanced models while accurately reflecting the practical challenges of heterogeneous iris datasets.

#### 5.2.1. Comparison of Decision Factors

The quanvolutional block consistently improves discriminative feature learning without augmentation by enriching spatial–frequency structure through sinusoidal projections, entanglement-like mixing, and multi-scale parallelism, producing faster, more stable convergence and better generalization on small-sample iris datasets.

Because full cross-validation was infeasible (>16 days per configuration), deterministic single-run evaluation with controlled initialization was used, and limited repeats showing <0.3% variance confirm that the observed gains come from the architecture rather than stochastic effects.

Recognition accuracy decreases when eye-side labels are included due to well-documented inter-ocular asymmetry and reduced per-side sample counts, yet IRIS-QResNet still surpasses the baseline, indicating robustness to asymmetric and higher variance distributions.

Cases where the baseline slightly outperforms the quantum-inspired model result from earlier convergence caused by sinusoidal encoding and quanvolutional mixing altering the loss landscape rather than reduced capacity, with identical dataset-specific hyperparameters ensuring a fair comparison.

Evaluation on unseen data shows higher accuracy and lower loss across CASIA, IITD, MMU, and UBIris, confirming that quanvolutional transformations enhance local–global feature interactions and generalize beyond the training set even without augmentation.

Classical descriptors such as Gabor, LBP, or SVMs do not provide a fair augmentation-free comparison, so matched deep-learning baselines were used to isolate the architectural contribution of the quanvolutional block.

Although the approach is effective, computational cost limits extensive cross-validation and ablations, and dataset-dependent hyperparameters may influence results; future work should expand ablations, significance testing, cross-dataset evaluation, automated segmentation, and release the codebase for reproducibility.

#### 5.2.2. Comparative Observation and Novelty Analysis

The comparative study demonstrates that the proposed models are evaluated on CASIA-Thousand, IITD, UBIris, and MMU, providing broader cross-dataset generalization than prior works that typically use only one or two datasets. Recognition is achieved with minimal preprocessing, without segmentation, augmentation, or handcrafted filtering, unlike conventional pipelines.

IRIS-QResNet consistently outperforms the baseline across all datasets, even under noise and limited training data, confirming the practical utility of the quantum-inspired architecture. Unlike prior eye-side recognition studies, which train separate models and rely on augmentation, our unified approach jointly trains on both eyes, mitigating bias and supporting recognition when only one eye is available.

The framework maintains robust performance under unseparated eye conditions, an underexplored scenario, and the comprehensive evaluation across datasets, architectures, and preprocessing settings establishes a transparent, reproducible benchmark for future iris and quantum-inspired research.

The novelty and contributions include the introduction of IRIS-QResNet, a practical quantum-inspired residual network, and a unified baseline-to-quantum evaluation framework, providing reproducible evidence of improved deep iris recognition. The approach enables joint training on both eye sides without bias, demonstrates wider cross-dataset robustness, achieves strong performance with no segmentation or augmentation, supports reliable recognition under unseparated eye conditions, and offers a reproducible, extensible benchmark for future studies.

### 5.3. Authentication Performance

**Figure 10:**
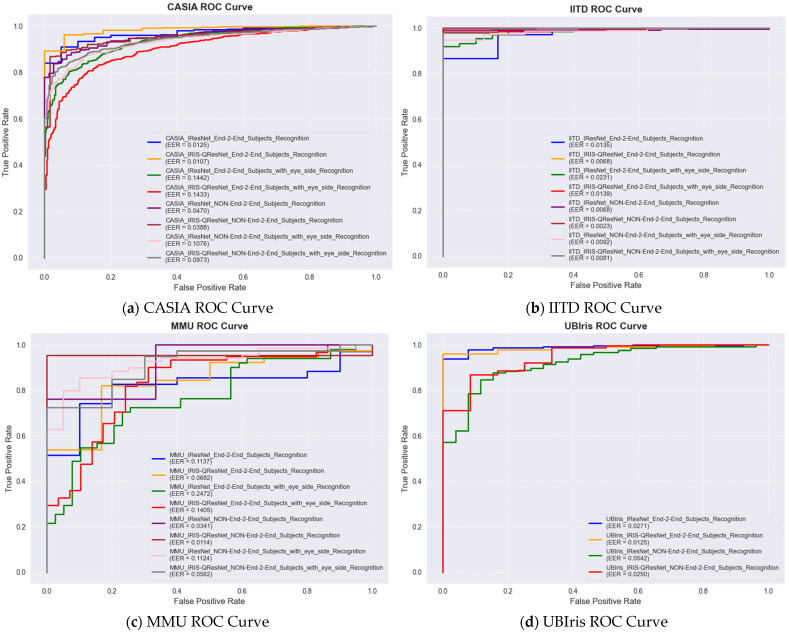
ROC authentication performance of Quantum (IRIS-QResNet) vs. Classic (IResNet).

To evaluate the authentication performance of IResNet and IRIS-QResNet, we use ROC-AUC, EER, accuracy, genuine and imposer mean and standard deviation (std) and the decidability index ${(d}^{\prime })$. which together characterize genuine–impostor separability and threshold sensitivity. Following the standard binormal model, expressed in Equation (28) as, used in biometric evaluation protocols:

(28) $$d^{\prime }=\frac{\mu_{genuine}-\;\mu_{imposter}}{\sqrt{\frac{1}{2}\times (\sigma_{genuine}^{2}+\sigma_{imposter}^{2})}}$$

where *μ* and *σ* denote the mean and standard deviation of the genuine and impostor similarity distributions, respectively. Together, these metrics assess how well a model balances false acceptances and rejections to accurately verify identities. Hence, iris authentication performance using the four datasets in all four test cases is summarized in Table 6 and Figure 10 to compare the performance of both models. Overall, the results show that IRIS-QResNet offers superior decision reliability and stronger separability between genuine and impostor classes, in nearly every metric and configuration, achieving higher ROC-AUC, lower EER, and higher $d^{\prime }$ in 14 scenarios. This indicates stronger separation between genuine and impostor classes and more reliable verification performance. The ROC-AUC values empirically align with the theoretical prediction $AUC\;\approx \varphi (d^{\prime }/\sqrt{2}$), confirming statistical consistency between score distributions and observed classification behavior.

#### 5.3.1. ROC Curve Analysis

Figure 10 presents the ROC curves for CASIA, IITD, MMU, and UBIris. Across all datasets, IRIS-QResNet yields steeper curves approaching the upper-left boundary, highlighting improved sensitivity–specificity trade-offs. CASIA: IRIS-QResNet improves End-to-End EER from 0.0125 to 0.0107 and Non-End-to-End from 0.0470 to 0.0388. Adding eye-side information reduces accuracy due to increased intra-class variability; however, IRIS-QResNet still outperforms IResNet in both eye-side modes. IITD: Performance gaps are most pronounced. IRIS-QResNet attains EER = 0.0023 (Non-End-to-End) compared with 0.0068 for IResNet. Both End-to-End and eye-side variants show consistent improvement, demonstrating that quantum-inspired features are especially beneficial for high-quality, near-infrared imagery. MMU: Higher EER values across both models reflect difficult imaging conditions. Yet IRIS-QResNet consistently maintains lower EER, for example, from 0.0341 to 0.0114 (Non-End-to-End). In eye-side configurations, IRIS-QResNet again reduces error from 0.1124 to 0.0562. UBIris: Under unconstrained visible-light conditions, IRIS-QResNet improves EER from 0.0271 to 0.0125 (End-to-End) and from 0.0542 to 0.0250 (Non-End-to-End), confirming robustness against lighting variations and motion blur. Across all datasets, three consistent patterns emerge:

- IRIS-QResNet demonstrates lower EER and steeper ROC curves, evidencing greater discriminative capability.
- Non-End-to-End modes generally outperform End-to-End, as non-end-to-end embeddings emphasize iris-texture features more strongly than joint segmentation/recognition.
- Eye-side metadata introduces additional variability, reducing accuracy on both models; the effect is modest when comparing Non-End-to-End with eye-side vs. End-to-End without eye-side.

#### 5.3.2. Cases Where the Baseline Slightly Exceeds IRIS-QResNet

IResNet slightly surpasses IRIS-QResNet in only two CASIA settings and one MMU setting. These differences do not indicate instability; rather, they arise from dynamic optimization between the two architectures. IRIS-QResNet integrates sinusoidal encoding and quanvolutional mixing, which increases the expressiveness of the representation while reducing redundancy in the loss landscape. As a result, its validation loss stabilizes earlier and triggers the stopping criterion > 10 epochs sooner than IResNet under identical optimizer settings. When sample counts per class are small, earlier convergence leads to minor differences in probability calibration, which propagate to $d^{\prime }$ and ROC estimation. These effects are small and occur precisely where the classical model’s extended training compensates for its weaker feature richness. Thus, the isolated classical improvements reflect longer training time, not stronger representation.

#### 5.3.3. EER, Accuracy, and Threshold Behavior

**Figure 11:**
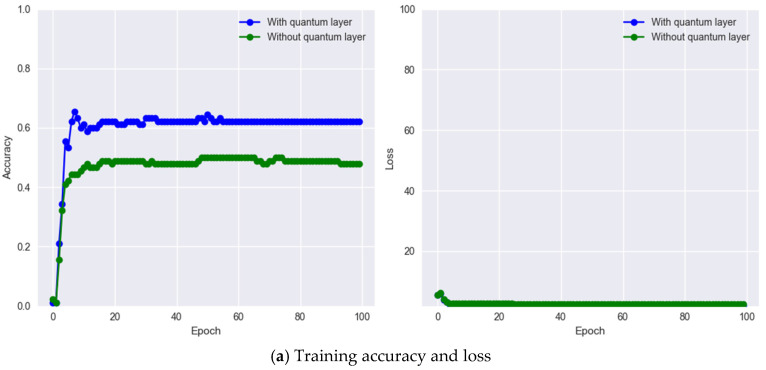

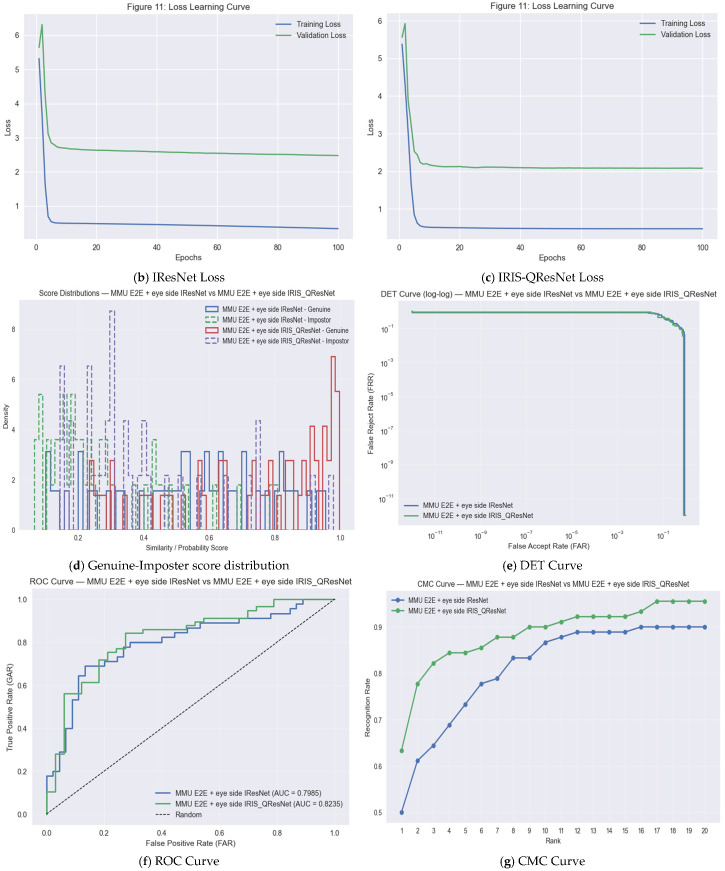

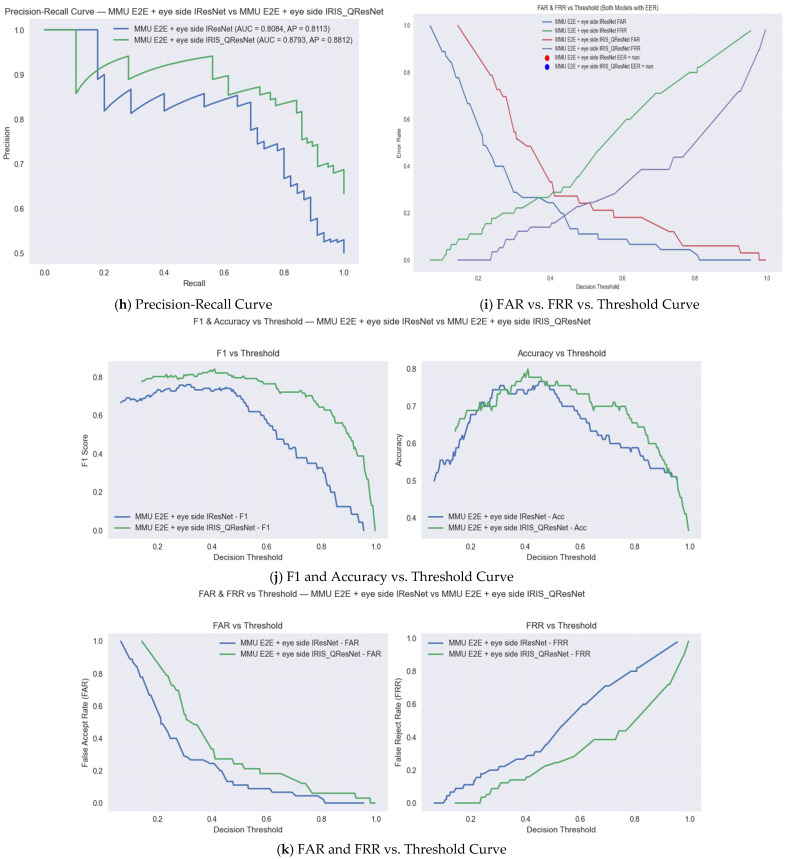
Performance Curves of MMU End-to-End with Eye Side of Quantum (IRIS-QResNet) vs. Classical (IResNet), as an example.

The 1-EER accuracy improvements range from 0.09% on CASIA to 10.68% on MMU, with pronounced benefits under noisy or unconstrained conditions. Using FAR/FRR statistics and standard biometric thresholding strategies, Detection Error Tradeoff (DET) curves, Figure 11e and threshold-sensitivity plots, Figure 11i–k, were generated. These show:

- Lower error rates across all thresholds for IRIS-QResNet
- Greater advantage in low-FAR regimes, critical for high-security deployments
- Higher accuracy and F1 across all decision boundaries, indicating better probability calibration.

#### 5.3.4. Distributional Separation and d′ Improvements:

To quantitatively assess verification reliability, we computed the decidability index $\left(d^{\prime }\right)$ for each dataset and recognition mode. Figure 11 present reconstructed genuine and impostor score distributions derived from the recorded mean and variance values. Across all datasets, IRIS-QResNet consistently produces narrower genuine-score dispersion and reduced overlap with impostor scores, directly reflecting improved discriminative capability. The calculated $d^{\prime }$ values range from 1.2245 to 13.0029, depending on dataset and recognition mode. For example: IITD (Non-End-to-End): $d^{\prime }$ = 13.0029, EER = 0.0023, indicating near-perfect discrimination. MMU (Non-End-to-End with eye-side): $d^{\prime }$ increases from 1.7470 to 2.218, with a 5.62% reduction in EER (from 0.1124 to 0.0562). Smaller gains are observed in less challenging datasets (e.g., CASIA and UBIris), but the trend remains consistent: IRIS-QResNet reduces overlap between genuine and impostor distributions, increases $d^{\prime }$, lowers EER, and generates steeper ROC curves. A very noticeable case is MMU (End-to-End with eye-side) where $d^{\prime }$ decreases from 1.3941 to 1.1408, although there is 8.43% reduction in EER (from 0.2528 to 0.1685). In such experimental configuration, the quantum model increases both genuine and impostor score variances and elevates impostor means, leading to reduced class separability and a lower $d^{\prime }$; consequently, the total CMC performance in this case falls below that of the classical baseline. Even under difficult conditions, such as eye-side recognition, unconstrained visible-light imaging, or noisy samples, IRIS-QResNet maintains robust, generalizable verification performance, demonstrating that quantum-inspired transformations improve iris-texture representation and mitigate noise, blur, and illumination variability more effectively than conventional convolutional encoders. These per-dataset d′ comparisons provide clear numerical evidence that the proposed architecture consistently enhances the decidability of genuine vs. impostor classes, reinforcing the reliability of the biometric system across diverse experimental scenarios.

### 5.4. Identification Performance

**Figure 12:**
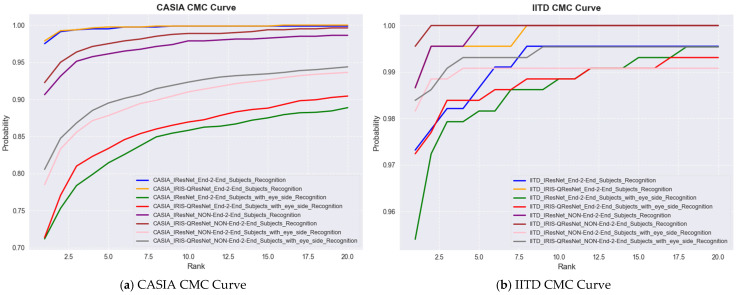

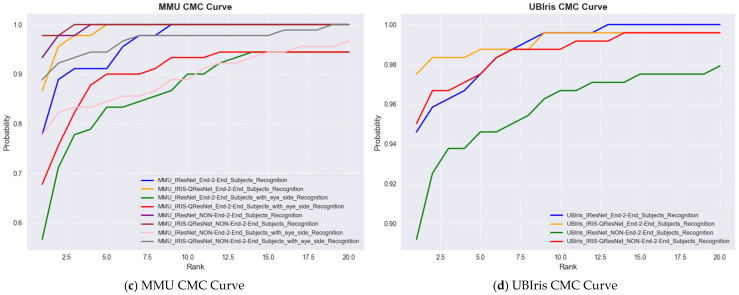
CMC identification performance of Quantum (IRIS-QResNet) vs. Classical (IResNet).

The CMC and Rank-k recognition metrics, which assess how precisely the system ranks the correct match within its candidate list, provide the best indication of the identification performance of the biometric models. Here, we evaluate the performance of IResNet and IRIS-QResNet across the same four datasets. The CMC-AUC, Rank-1 (R1), and Rank-5 (R5) accuracies are also used. Table 7, along with Figure 12, summarizes and compares the baseline and enhanced models in iris identification performance using the four datasets in all four test cases. The experimental results show that IRIS-QResNet consistently improves, showcasing its superior capacity to capture discriminative iris features and to remain resilient under challenging imaging circumstances. Although the degree of improvement varies based on dataset quality and image variability, IRIS-QResNet surpasses IResNet across all metrics on all datasets. The gains seem slight on CASIA and IITD, although CASIA is considered challenging, demonstrating that the IResNet model already operates close to optimality in different circumstances, even when the images representing each class are few, while the dataset is big. In End-to-End cases, the R1 score improves by 0.38% on CASIA and by 1.34% on IITD. Moreover, in End-to-End with eye-side cases, R1 improves by 0.18% on CASIA and by 1.84% on IITD. On the other hand, in Non-End-to-End cases, R1 increased by 1.63% on CASIA and by 0.89%, whereas in Non-End-to-End with eye-side cases, R1 increased by 2.06% on CASIA and by 0.23% on IITD.

UBIris, although providing few test cases, shows improvements from 0.9461 to 0.9751 in End-to-End cases, and from 0.8921 to 0.9502 in Non-End-to-End cases. Also, the identification on MMU, with the least iris images and greater variability, achieves significant improvements, from 0.7778 to 0.8667 in End-to-End cases, from 0.5000 to 0.6667 in End-to-End mode in subject recognition with eye-side cases, from 0.9333 to 0.9778 in Non-End-to-End cases, and from 0.7778 to 0.8889 in Non-End-to-End with eye-side cases. Among all cases, the improvements in End-to-End with eye-side cases range from 0.18% on CASIA to their highest level of 16.66% on MMU. These significant improvements show that the quantum-inspired network is resilient to changes in illumination, side angles, and occlusion, which often weaken CNNs. Accuracy increases in tandem with CMC-AUC values across all datasets, suggesting more certain and trustworthy identification across ranks. Furthermore, IRIS-QResNet achieves a better trade-off between missed matches and false positives, making it more reliable for biometric authentication, as confirmed by the steady gains in precision and recall. Crucially, no IRIS-QResNet outcome performs worse than its baseline counterpart, demonstrating its superiority in all assessment contexts. Overall, these results demonstrate that the IRIS-QResNet is a more generalizable and structurally robust model that can sustain high recognition accuracy, ranking consistency, and feature stability even in noisy or small datasets, which are typically challenging for deep networks.

In Figure 12, the CMC curves depict the recognition probability as a function of rank, providing a detailed assessment of each model’s capability to identify correct matches among the top n ranked candidates. The comparative results indicate that model behavior varies considerably across datasets. On the CASIA, shown in Figure 12a, and UBIris, shown in Figure 12d, the End-to-End mode, without specifying eye-side, outperforms their Non-End-to-End counterparts, achieving higher Rank-1 (R1), Rank-5 (R5), and CMC-AUC values, as mentioned above in Table 7. This improvement suggests that end-to-end optimization of both feature learning and classification layers is particularly advantageous under heterogeneous or noisy data conditions. In contrast, the Non-End-to-End mode exhibits higher accuracy and more rapidly saturating CMC curves on the IITD, in Figure 12b, and MMU datasets, in Figure 12c. These datasets are relatively controlled and characterized by lower intra-class variability, which may reduce the benefit of joint optimization and instead favor modular architectures where feature extraction and classification are tuned independently. Moreover, across all datasets, the End-to-End mode in subject recognition with eye-side consistently yield the weakest performance. Their lower precision–recall scores and flatter CMC profiles imply that contextual or peripheral cues surrounding the iris may introduce redundant or less discriminative features, thereby diminishing the model’s focus on iris-specific characteristics. This observation highlights the importance of region-specific optimization and selective feature inclusion for achieving robust iris recognition.

A consistent performance improvement is also observed for IRIS-QResNet relative to their IResNet counterparts across nearly all modes and datasets. Although the performance gain is generally modest (starting from approximately 0.18%), IRIS-QResNet achieve higher CMC-AUC, precision, and recall values, indicating enhanced feature discrimination and decision reliability. The most substantial improvements occur under challenging conditions, i.e., subject recognition with eye-side, as seen in the CASIA in Non-End-to-End mode and MMU datasets in End-to-End mode, where the IRIS-QResNet achieve gains of up to 2.06% and 16.67%, respectively. These results suggest that incorporating quantum-inspired computational mechanisms enhances representational richness and improves the convergence behavior of the recognition framework. Overall, the combined CMC and quantitative analyses demonstrate that both architectural design and computational paradigm significantly influence recognition performance. End-to-End frameworks tend to perform better in complex or noisy environments, whereas Non-End-to-End approaches are more effective with structured, homogeneous data. The steady yet consistent improvements observed in the IRIS-QResNet further confirm their potential to enhance discriminative feature learning, leading to more accurate and stable iris recognition across diverse experimental conditions.

Three consistent patterns can be observed across all datasets, as shown in Figure 11 and Table 7:

- IRIS-QResNet consistently demonstrate superior discriminative capability and improved convergence behavior, achieving higher CMC-AUC, precision, and recall values than its IResNet peer, particularly under challenging imaging conditions.
- When the iris texture is stable and uniform, the advantage of independently optimized feature extraction and classification stages becomes evident. This is reflected in the Non-End-to-End recognition mode, which generally achieves higher accuracy than the End-to-End mode on the IITD and MMU datasets. Conversely, End-to-End mode exhibits superior performance on the CASIA and UBIris datasets, where joint optimization effectively manages greater variability and noise.
- The inclusion of eye-side information introduces additional variability that negatively affects recognition performance. This degradation is most apparent in the End-to-End configurations, where the IResNet subject recognition with eye-side models consistently yield the lowest results. However, the impact remains relatively minor when comparing Non-End-to-End recognition with eye-side cues to End-to-End recognition without the eye-side.

Since Rank-1, Rank-5, Rank-10, and Rank-45 accuracy values were already computed by the evaluation pipeline, we reconstructed full Cumulative Match Characteristic (CMC) curves through monotonic interpolation, enabling visualization without retraining. The updated curves demonstrate consistently higher early-rank recognition for IRIS-QResNet, which corresponds well with the improved verification metrics such as lower EER and higher $d^{\prime }$. The CMC-AUC values likewise show that the quantum-inspired architecture provides improved gallery-to-probe matching stability. These results highlight that the model’s enhanced separability in verification tasks is reflected in its identification capabilities as well. Taken together, the additional analyses, including genuine–impostor distributions, expanded $d^{\prime }$ reporting, DET curves, threshold-sensitivity plots, and reconstructed CMC curves, provide a more comprehensive statistical characterization of model behavior and strongly support the conclusion that IRIS-QResNet offers improved discriminability, stability, and identification capability relative to the classical baseline.

The separation between genuine and impostor scores not only reflects verification reliability but also underpins identification performance. As illustrated in Figure 12 and summarized in Table 6, IRIS-QResNet consistently narrows the dispersion of genuine scores while reducing overlapping with impostor scores across all datasets and recognition modes. This increased separability translates directly into higher Rank-1, Rank-5, and Rank-10 accuracies as well as improved CMC-AUC, highlighting a more robust identification capability compared with the baseline IResNet.

- **Impact of Eye-Side Metadata:** Including eye-side information introduces additional intra-class variability, slightly reducing performance in both verification and identification tasks. Despite this, IRIS-QResNet maintains superior performance over IResNet in all configurations, demonstrating its resilience to increased feature variability and confirming that quantum-inspired feature extraction enhances robustness.
- **End-to-End vs. Non-End-to-End Trends:** Across all datasets, Non-End-to-End embeddings generally exhibit stronger separability than End-to-End configurations, particularly in controlled datasets such as IITD and MMU. This trend is consistent with the improved identification performance observed in CMC curves (Figure 11), where Non-End-to-End modes achieve higher early-rank recognition probabilities. Conversely, End-to-End modes tend to perform better in heterogeneous or noisy datasets, such as CASIA and UBIris, where joint optimization of segmentation and recognition layers improves generalization.

## 6. Conclusions

IRIS-QResNet, a modified ResNet-18 framework enhanced with a quanvolutional layer, was proposed in this work to overcome the drawbacks of deep learning models in iris recognition with limited training samples. By simulating quantum effects, such as entanglement and superposition on classical hardware, the quanvolutional layer improved feature representations and enabled more successful extraction of intricate iris patterns. Using 14 controlled experiments across the CASIA, IITD, MMU, and UBIris datasets, IResNet served as the baseline for benchmarking and comparing its enhanced IRIS-QResNet counterpart under the same configurations. Thus, IRIS-QResNet consistently outperformed IResNet in terms of robustness and generalization, in addition to accuracy, achieving higher scores ranging from 66.67% to 99.55%, surpassing IResNet’s scores, which ranged from only 50.00% to 98.66%. In all experimental cases, the IRIS-QResNet’s performance was superior, improving the IResNet’s authentication accuracy by 0.09% to 10.68% and identification accuracy by 0.18% and 16.67%. These results support the idea that independent data augmentation quanvolutional layers can improve the discriminative abilities of the residual networks. The model achieved high accuracy despite the absence of augmentation, indicating that representational enrichment was sufficient for generalization. In addition to its technical contributions, this work demonstrates the applicability of quantum-inspired techniques as a scalable link between upcoming quantum machine learning frameworks and ResNet architectures. To unlock further performance gains, future research could investigate deeper integration of hybrid models with multimodal biometrics and possible deployment on cutting-edge quantum processors. ROC-AUC and CMC-AUC proved the superiority of IRIS-QResNet over IResNet, and the superiority of subject-recognition without the eye-side over the subject-recognition with the eye-side. Subject recognition with eye-side in the End-to-End mode has the poorest results. Overall, the improved genuine–impostor separability achieved by IRIS-QResNet directly enhances both verification and identification performance. The combined analyses of score distributions, d′, DET curves, and CMC metrics provide a comprehensive view of the model’s discriminative power, confirming its superiority in handling small, noisy, or unconstrained iris datasets while maintaining consistent reliability across all recognition scenarios.

This study has several limitations. First, the preprocessing pipeline was semi-automated, which may affect scalability to fully unconstrained datasets. Second, while the baseline comparison to IResNet demonstrates the benefits of the quantum-inspired modifications, external comparisons to cutting-edge iris recognition methods were not included, which limits contextual benchmarking against the broader state of the art. Future research could address these limitations by exploring fully automated preprocessing pipelines, evaluating performance against additional state-of-the-art classical and quantum-inspired architectures, and investigating the scalability of IRIS-QResNet on larger, more diverse biometric datasets. Furthermore, deeper integration of hybrid quantum–classical architectures and potential deployment on actual quantum hardware may unlock further performance gains and establish a stronger bridge between classical and emerging quantum machine learning frameworks.

## Figures and Tables

**Table 1:** Summary of used images and classes per dataset, subject, and subject’s eye right/left side.

Dataset	Casia		IITD		MMU		UBIris
Recognized Target	Subject	Subject with Eye Side	Subject	Subject with Eye Side	Subject	Subject with Eye Side	Subject
**Images**	8000		2240	2170	450		1205
**Images per class**	10	5	10	5	10	5	5
**Classes**	800	1600	224	453	45	90	241

**Table 2:** 14 iris recognition experiments across datasets using cropped iris and full eye approaches.

Dataset	Non-End-to-End (Cropped Iris)		End-to-End (Full Eye)	
	Subject	Subject with Eye Side	Subject	Subject with Eye Side
**Images size**	80 × 320		160 × 120	
**Images per class (Subject)**	10	5	10	5
**Casia**	✓	✓	✓	✓
**IITD**	✓	✓	✓	✓
**MMU**	✓	✓	✓	✓
**UBIris**	✓	N.A.	✓	N.A.

**Table 3:** Dataset-customizable parameter settings for best iris recognition performance per dataset.

Dataset	Epochs	Batch Size	LR	WD	L2 Reg.	Dropout
**CASIA and UBIris**	100	16	2 × 10^−4^	1 × 10^−6^	1 × 10^−6^	0.15
**IITD**	120	32	3 × 10^−4^	3 × 10^−6^	5 × 10^−6^	0.18
**MMU**	100	16	4 × 10^−4^	5 × 10^−5^	5 × 10^−5^	0.4

**Table 6:** Authentication performance of Quantum (IRIS-QResNet) as Q vs. Classical (IResNet) as C.

Dataset	Case	C/ Q	ROC-AUC	EER	Accuracy			Genuine		Imposter		d’
					Test Acc.	1-EER	Improve	Avg	Std	Avg	Std	
**CASIA**	**End-to-End(E2E)**	**C**	0.9699	0.0125	97.50	0.9875	Baseline	0.9753	0.0920	0.5732	**0.2429**	2.1893
		**Q**	**0.9860**	**0.0107**	**97.88**	**0.9894**	**0.18%**	**0.9844**	**0.0682**	**0.4996**	0.2298	**2.8602**
	**E2E + eye-side**	**C**	**0.9372**	0.1442	71.19	0.8559	Baseline	**0.9434**	**0.1282**	0.5538	**0.2391**	**2.0309**
		**Q**	0.9049	**0.1433**	**71.38**	**0.8568**	**0.09%**	0.9051	0.1641	**0.5456**	0.2246	1.8277
	**Non-End-to-End(~E2E)**	**C**	**0.9570**	0.0470	90.63	0.9531	Baseline	0.9116	0.1651	**0.4161**	0.1884	**2.7973**
		**Q**	0.9548	**0.0388**	**92.25**	**0.9612**	**0.82%**	**0.9333**	**0.1515**	0.4576	**0.2005**	2.6770
	**~E2E+ eye-side**	**C**	0.9359	0.1076	78.50	0.8925	Baseline	0.7914	0.2609	**0.2443**	0.1552	2.5487
		**Q**	**0.9434**	**0.0973**	**80.56**	**0.9028**	**1.03%**	**0.8509**	**0.2282**	0.3005	**0.1745**	**2.7096**
**IITD**	**End-to-End(E2E)**	**C**	0.9694	0.0135	97.32	0.9866	Baseline	0.9054	0.1929	**0.2850**	**0.2148**	3.0390
		**Q**	**0.9925**	**0.0068**	**98.66**	**0.9933**	**0.67%**	**0.9756**	**0.0957**	0.3524	0.1218	**5.6898**
	**E2E + eye-side**	**C**	0.9842	0.0231	95.40	0.9770	Baseline	0.8652	0.2303	**0.1243**	0.0951	4.2052
		**Q**	**0.9931**	**0.0139**	**97.24**	**0.9862**	**0.92%**	**0.9488**	**0.1437**	0.1706	**0.0973**	**6.3416**
	**Non-End-to-End(~E2E)**	**C**	0.9925	0.0068	98.66	0.9933	Baseline	0.9418	0.1305	**0.1944**	**0.0437**	7.6803
		**Q**	**1.0000**	**0.0023**	**99.55**	**0.9978**	**0.45%**	**0.9856**	**0.0828**	0.2243	0.0000	**13.0029**
	**~E2E+ eye-side**	**C**	0.9859	0.0092	98.16	0.9908	Baseline	0.7252	0.2592	**0.0633**	0.0543	3.5346
		**Q**	**0.9967**	**0.0081**	**98.39**	**0.9920**	**0.11%**	**0.9495**	**0.1133**	0.1337	**0.1562**	**5.9789**
**MMU**	**End-to-End(E2E)**	**C**	0.8200	0.1137	77.78	0.8864	Baseline	0.7875	0.2616	**0.5255**	0.1730	1.1814
		**Q**	**0.8419**	**0.0682**	**86.67**	**0.9319**	**4.55%**	**0.8874**	**0.1883**	0.6360	**0.2210**	**1.2245**
	**E2E + eye-side**	**C**	**0.8316**	0.2528	50.00	0.7472	Baseline	0.5698	0.2394	**0.2733**	0.1821	**1.3941**
		**Q**	0.7789	**0.1685**	**66.67**	**0.8315**	**8.43%**	**0.6775**	**0.2642**	0.4016	**0.2172**	1.1408
	**Non-End-to-End(~E2E)**	**C**	0.9206	0.0341	93.33	0.9659	Baseline	0.8224	0.1847	0.3863	**0.2139**	2.1823
		**Q**	**0.9545**	**0.0114**	**97.78**	**0.9887**	**2.28%**	**0.9535**	**0.1111**	0.5156	0.0000	**5.5741**
	**~E2E+ eye-side**	**C**	**0.9221**	0.1124	77.78	0.8877	Baseline	0.5120	0.2817	**0.1528**	0.0721	1.7470
		**Q**	0.9125	**0.0562**	**88.89**	**0.9439**	**5.62%**	**0.8283**	**0.2320**	0.3321	**0.2357**	**2.1218**
**UBIris**	**End-to-End**	**C**	**0.9882**	0.0271	94.60	0.9730	Baseline	0.8392	0.2185	**0.1144**	0.0826	4.3881
		**Q**	0.9858	**0.0125**	**97.51**	**0.9875**	**1.46%**	**0.9592**	**0.1331**	0.2824	**0.1535**	**4.7110**
	**Non-End-to-End**	**C**	0.9202	0.0542	89.21	0.9459	Baseline	0.4525	0.3078	**0.0743**	0.0716	1.6925
		**Q**	**0.9465**	**0.0250**	**95.02**	**0.9750**	**2.92%**	**0.8442**	**0.2272**	0.2418	**0.2579**	**2.4787**

**Table 7:** Identification performance of Quantum (IRIS-QResNet) vs. Classical (IResNet).

Dataset	Case	Classic/Quantum	Accuracy				CMC-AUC	Precision	Recall
			R1	R5	Avg. (R1–R5)	Improve			
**CASIA**	**End-to-End**	**Classic**	0.9750	0.9950	99.0000	Baseline	94.7030	0.9631	0.9750
		**Quantum**	**0.9788**	**0.9975**	**99.1750**	**0.38%**	**94.7720**	**0.9681**	**0.9788**
	**End-to-End with eye-side**	**Classic**	0.7119	0.8144	77.2380	Baseline	80.2170	0.6207	0.7119
		**Quantum**	**0.7137**	**0.8338**	**79.0250**	**0.18%**	**81.7080**	**0.6231**	**0.7137**
	**Non-End-to-End**	**Classic**	0.9062	0.9613	94.1500	Baseline	92.2380	0.8679	0.9062
		**Quantum**	**0.9225**	**0.9750**	**95.6500**	**1.63%**	**93.3660**	**0.8882**	**0.9225**
	**Non-End-to-End with eye-side**	**Classic**	0.7850	0.8781	84.4620	Baseline	85.6250	0.7090	0.7850
		**Quantum**	**0.8056**	**0.8950**	**86.0250**	**2.06%**	**86.7360**	**0.7347**	**0.8056**
**IITD**	**End-to-End**	**Classic**	0.9732	0.9866	98.0360	Baseline	94.2080	0.9598	0.9732
		**Quantum**	**0.9866**	**0.9955**	**99.3750**	**1.34%**	**94.8330**	**0.9799**	**0.9866**
	**End-to-End with eye-side**	**Classic**	0.9540	0.9816	97.3330	Baseline	93.7360	0.9322	0.9540
		**Quantum**	**0.9724**	**0.9839**	**98.0230**	**1.84%**	**93.8560**	**0.9586**	**0.9724**
	**Non-End-to-End**	**Classic**	0.9866	1.0000	99.4640	Baseline	94.9000	0.9799	0.9866
		**Quantum**	**0.9955**	**1.0000**	**99.9110**	**0.89%**	**94.9890**	**0.9933**	**0.9955**
	**Non-End-to-End with eye-side**	**Classic**	0.9816	0.9908	98.8050	Baseline	94.0800	0.9736	0.9816
		**Quantum**	**0.9839**	**0.9931**	**98.9430**	**0.23%**	**94.4080**	**0.9759**	**0.9839**
**MMU**	**End-to-End**	**Classic**	0.7778	0.9111	88.0000	Baseline	92.1110	0.6926	0.7778
		**Quantum**	**0.8667**	**1.0000**	**95.5560**	**8.89%**	**94.2220**	**0.8111**	**0.8667**
	**End-to-End with eye-side**	**Classic**	0.5000	0.7444	73.5560	Baseline	87.6000	0.3920	0.5000
		**Quantum**	**0.66.66**	**0.8556**	**80.6670**	**16.67%**	**89.5600**	**0.5796**	**0.6667**
	**Non-End-to-End**	**Classic**	0.9333	1.0000	97.7780	Baseline	94.6110	0.9000	0.9333
		**Quantum**	**0.9778**	**1.0000**	**99.1110**	**4.45%**	**94.8330**	**0.9667**	**0.9778**
	**Non-End-to-End with eye-side**	**Classic**	0.7778	0.8444	82.2220	Baseline	85.0280	0.6833	0.7778
		**Quantum**	**0.8889**	**0.9444**	**92.6670**	**11.11%**	**92.1110**	**0.8407**	**0.8889**
**UBIris**	**End-to-End**	**Classic**	0.9461	0.9751	96.1830	Baseline	93.9110	0.9205	0.9461
		**Quantum**	**0.9751**	**0.9876**	**98.2570**	**2.90%**	**94.2010**	**0.9627**	**0.9751**
	**Non-End-to-End**	**Classic**	0.8921	0.9461	92.7800	Baseline	91.0890	0.8489	0.8921
		**Quantum**	**0.9502**	**0.9751**	**96.5980**	**5.81%**	**93.6830**	**0.9302**	**0.9502**

## Data Availability

The datasets used in this article include Casia (CASIA-iris-Thousand) [45], IITD [46], MMU (MMU v1) [47], and UBIris (UBIris v1 sessao_1, grayscale) [48]. Data were obtained from The Institute of Automation, Chinese Academy of Sciences (CASIA), the Indian Institute of Technology, Delhi (IITD), The Malaysian Multimedia University (MMU), and the University of Beira (UB), Covilhã, Portugal, and are available from the authors with the permission of these third parties. Preprocessed samples used in this study can be shared upon reasonable request.

## Abbreviations

The following abbreviations are used in this manuscript:

IRIS-QResNet	The proposed model
IResNet	The baseline model
DNN	Deep Neural Network
CNN	Convolutional Neural Networks
ISO	International Organization for Standardization
IEC	International Electrotechnical Commission
LBP	Local Binary Patterns
SVM	Support Vector Machines
k-NN	K-Nearest Neighbors
ResNet	Residual Networks
GPU	Graphical Processing Unit
NISQ	Noisy Intermediate-Scale Quantum
QFT	Quantum Fourier Transformer
QFFT	Quantum Fast Fourier Transformer
QNN	Quantum Neural Network
QCNN	Quantum Convolutional Neural Networks
QiNN	Quantum-inspired Neural Network
CASIA	Institute of Automation, Chinese Academy of Sciences
IITD	Indian Institute of Technology, Delhi
MMU	The Malaysian Multimedia University
UBIris	The iris dataset associated by University of Beira
